# Phase Transformation Dynamics in Sulfate-Loaded Lanthanide Triphosphonates. Proton Conductivity and Application as Fillers in PEMFCs

**DOI:** 10.1021/acsami.1c01441

**Published:** 2021-03-25

**Authors:** Inés R. Salcedo, Rosario M. P. Colodrero, Montse Bazaga-García, M. López-González, Carmen del Río, Konstantinos Xanthopoulos, Konstantinos D. Demadis, Gary B. Hix, Aleksandra D. Furasova, Duane Choquesillo-Lazarte, Pascual Olivera-Pastor, Aurelio Cabeza

**Affiliations:** ‡ Departamento de Química Inorgánica, Cristalografía y Mineralogía, Universidad de Málaga, Campus de Teatinos s/n, Málaga-29071, Spain; § Instituto de Ciencia y Tecnología de Polímeros (ICTP-CSIC), Juan de la Cierva 3, Madrid-28006, Spain; ¥ Crystal Engineering, Growth and Design Laboratory, Department of Chemistry, University of Crete, Heraklion, Crete, GR-71003, Greece; # School of Sciences, University of Wolverhampton, Wulfruna Street, Wolverhampton WV1 1LY, United Kingdom; £ Department of Physics and Engineering, ITMO University, St. Petersburg 197101, Russia; ⊥ Laboratorio de Estudios Cristalográficos, IACT (CSIC-UGR), Avda. de las Palmeras 4, 18100 Armilla, Granada , Spain

**Keywords:** metal phosphonate, proton conductivity, composite membranes, Nafion, PEMFCs

## Abstract

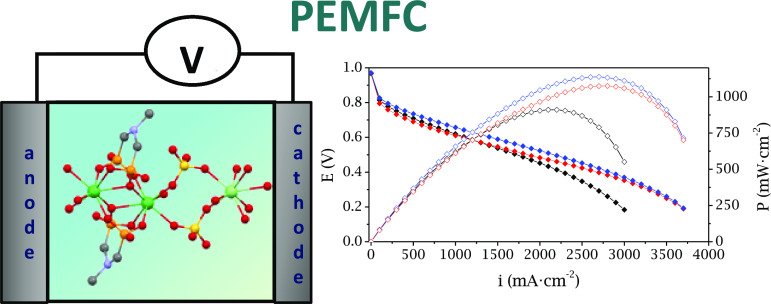

Phase transformation dynamics and proton conduction properties are reported for cationic layer-featured coordination polymers derived from the combination of lanthanide ions (Ln^3+^) with nitrilo-tris(methylenephosphonic acid) (H_6_NMP) in the presence of sulfate ions. Two families of materials are isolated and structurally characterized, i.e., [Ln_2_(H_4_NMP)_2_(H_2_O)_4_](HSO_4_)_2_·*n*H_2_O (Ln = Pr, Nd, Sm, Eu, Gd, Tb, Er, Yb; *n* = 4–5, **Series I**) and [Ln(H_5_NMP)]SO_4_·2H_2_O (Ln = Pr, Nd, Eu, Gd, Tb; **Series II**). Eu/Tb bimetallic solid solutions are also prepared for photoluminescence studies. Members of families **I** and **II** display high proton conductivity (10^–3^ and 10^–2^ S·cm^–1^ at 80 °C and 95% relative humidity) and are studied as fillers for Nafion-based composite membranes in PEMFCs, under operating conditions. Composite membranes exhibit higher power and current densities than the pristine Nafion membrane working in the range of 70–90 °C and 100% relative humidity and with similar proton conductivity.

## Introduction

Up to now, Nafion has been the benchmark of polymer electrolytes. However, new advances are desirable to facilitate enhanced performance for proton exchange membrane fuel cells (PEMFCs).^1^ Among the various strategies implemented,^2,3^ incorporating or doping Nafion membranes with inorganic nanoparticles has beneficial effects on thermal stability, water uptake and/or management, and proton conductivity. In addition, Nafion-mixed membranes can provide higher power densities than the pristine Nafion membranes.^4−7^

Coordination polymers (CPs) exhibit high crystallinity,^8^ precise proton transfer pathways,^9^ and a wide variety of structural architectures that depend on the metal center^10^ and/or the organic linker,^11^ which make them attractive as potential fillers for Nafion-mixed membranes. Among the plethora of CPs,^12−14^ metal phosphonate-based proton conductors hold a prominent position.^15−19^ In these, the organic linker contains one or more phosphonate groups (−PO_3_H*_x_*^–(2–*x*)^, where *x* = 0, 1, or 2, depending on the deprotonation degree). Moreover, organic phosphonic acid frameworks (phosphonate HOFs), with supramolecular architectures, are attractive materials due to their porosity and conductive behavior, including proton conductivity.^20^

Additional advantages of metal phosphonates in comparison to other CPs are (a) increased hydrolytic stability of the P–C and M–O bonds,^21^ (b) enhanced thermal stability,^22^ (c) variable structures with well-defined proton conduction pathways,^23^ (d) acidic sites on the ligands,^24^ (e) water molecules (either metal-coordinated or in the lattice) that commonly act as proton carriers,^25^ and (f) the ability to host anions or cations (either in channels or in the interlayer space) that may augment proton conduction.^26,27^ In addition, derivatizing CPs with strongly acidic groups has shown to be a highly efficient strategy for enhancing proton conductivity.^28,29^

Recently, results from our collaborative effort have been published on the use of layered lanthanide CPs utilizing a phosphonate derivative of taurine (namely, 2-[bis(phosphonomethyl)amino]ethanosulfonic acid), which assists in maintaining the proton conductivity of Nafion membranes at least up to 90 °C and perform satisfactorily in PEMFC single cells.^30^

Combined with divalent transition metals or lanthanide-ions, nitrilo-tris(methylenephosphonic acid) (H_6_NMP) yields acidic bidimensional solids.^31^ In contrast to the divalent metal derivatives, which form neutral layers,^31^ lanthanide derivatives present both neutral^32^ and positively charge layers with interesting properties as proton conductors, catalysts, and photoluminescent materials.^33−35^ Moreover, the latter undergo dynamic structural transformation, as observed for [Ln(H_4_NMP)(H_2_O)_2_](Cl)·2H_2_O [Ln = La^3+^, Pr^3+^, Sm^3+^, Eu^3+^, Gd^3+^, Tb^3+^, Dy^3+^, Ho^3+^).^33−35^ Interestingly, the Gd^3+^ derivative was found to convert, at high humidity and temperature, into a chloride-free compound, which demonstrated substantially enhanced proton conductivity, up to 0.51 S·cm^–1^.^35^

Herein, we present a detailed study in order to access to Ln-H*_x_*NMP derivatives containing the proton-enhancing sulfate ion. Incorporating sulfate species into the framework of CPs can be an effective way of enhancing proton conductivity.^36^ Hence, we report the syntheses and structural features of several compounds as members of the growing family of lanthanide-sulfate-nitrilo(trismethylenephosphonates). These compounds display high proton conductivity values between 10^–3^ and 10^–2^ S·cm^–1^ at 80 °C and 95% RH. Selected materials have also been studied as fillers for the preparation of Nafion-mixed membranes, and their responses in proton exchange membrane fuel cells (PEMFCs) have been established under operating conditions. Preliminary studies show that the resulting composite membranes exhibit similar proton conductivity behavior and, in some cases, higher power and current densities than the pristine Nafion membrane working in the range of 70–90 °C and 100% RH.

## Experimental Section

### Materials and Instrumentation

Hydrated lanthanide nitrate or chloride reagents and H_6_NMP (50% w/w solution in water as acid) were purchased from Sigma-Aldrich. Sulfuric acid was from BDH Prolabo. In-house deionized (DI) water was used for all synthesis. Nafion 1100 EW dispersion 20 wt % in a mixture of water and alcohols was purchased from Ion Power. Isopropanol was obtained from Merck & Co. Elemental analyses (C, H, N, S) were measured on a TruSpec Macro CHN-S analyzer. Thermogravimetric analysis (TGA) data were recorded on an SDT-Q600 analyzer from TA Instruments. The temperature varied from RT to 900 °C at a heating rate of 10 °C/min. Measurements were carried out on samples in open platinum crucibles under a flow of air or N_2_. Lanthanide and phosphorus contents in solids were determined by inductively coupled plasma mass spectrometry analysis (ICP-MS).

### Synthesis

Two families of sulfate-containing polymorphs were isolated depending on the specific synthesis conditions, starting from two solutions separately prepared and then mixed. The lanthanide salt (0.46 mmol) was dissolved in DI water (10 mL). The ligand solution was obtained upon dilution of H_6_NMP (2.30 mmol, 1.5 mL) in DI water (20 mL). Compounds **Series I**, [Ln_2_(H_4_NMP)_2_(H_2_O)_4_](HSO_4_)_2_·*n*H_2_O (Ln = Pr^3+^, Nd^3+^, Sm^3+^, Eu^3+^, Gd^3+^, Tb^3+^, Er^3+^, Yb^3+^; *n* = 4–5), or **Series II**, Ln[(HNMP)]SO_4_·2H_2_O (Ln = Pr^3+^, Nd^3+^, Eu^3+^, Gd^3+^, Tb^3+^), were obtained by crystallization at 25 °C for several days in the presence of H_2_SO_4_ (95%) in specific amounts. Compounds of **Series I** required the addition of a volume of H_2_SO_4_ (95%) ranging between 0.5 and 2.5 (Eu^3+^) to 3.5 (Pr^3+^) mL. Higher volumes of H_2_SO_4_ (95%) up to 5 mL led to **Series II** compounds. All attempts to extend the synthesis beyond the Tb^3+^ derivative were unsuccessful. Compound Pr-I* was synthesized as follows: 0.25 mmol of Pr(NO_3_)_3_·6H_2_O was dissolved in 5 mL of DI water and was then added to 0.217 mL of H_6_NMP aqueous solution (0.47 mmol H_6_NMP). Finally, 0.5 mL of H_2_SO_4_ (95%) was added to the solution mixture. This solution was left to crystallize in a glass test tube at RT for several days. The obtained solids were filtered off, washed with DI water, and air-dried. Typical yields for compounds of **Series I** ranged between 33 and 84%, based on the metal, for Er^3+^ and Tb^3+^ derivatives, respectively.

Elemental composition (wt %) for compounds of **Series I**: Anal. calcd for Pr_2_C_6_H_38_N_2_O_34_P_6_S_2_: C 5.94, H 3.15, N 2.31, S 5.28; found C 5.87, H 2.77, N 2.36, S 5.10. Anal. calcd for Nd_2_C_6_H_38_N_2_O_34_P_6_S_2_: C 5.90, H 3.14, N 2.29, S 5.25; found C 5.88, H 2.99, N 2.32, S 4.98. Anal. calcd for Sm_2_C_6_H_38_N_2_O_34_P_6_S_2_: C 5.84, H 3.11, N 2.27, S 5.20; found C 5.66, H 3.17, N 2.27, S 5.75. Anal. calcd for Eu_2_C_6_H_38_N_2_O_34_P_6_S_2_: C 5.83, H 3.09, N 2.27, S 5.19; found C 5.46, H 2.91, N 2.25, S 5.02. Anal. calcd for Gd_2_C_6_H_38_N_2_O_34_P_6_S_2_: C 5.78, H 3.07, N 2.25, S 5.14; found C 5.58, H 3.00, N 2.26, S 5.40. Anal. calcd for Tb_2_C_6_H_38_N_2_O_34_P_6_S_2_: C 5.76, H 3.06, N 2.24, S 5.13; found C 5.58, H 3.00, N 2.27, S 5.18. Anal. calcd for Er_2_C_6_H_40_N_2_O_35_P_6_S_2_: C 5.61, H 3.14, N 2.18, S 4.99; found C 5.24, H 2.85, N 3.13, S 5.42. Anal. calcd for Yb_2_C_6_H_40_N_2_O_35_P_6_S_2_: C 5.56, H 3.11, N 2.16, S 4.95; found C 5.27, H 3.37, N 2.20, S 4.49.

Elemental composition (wt %) for compounds of **Series II**: Anal. calcd for PrC_3_H_15_NO_15_P_3_S: C 6.31, H 2.65, N 2.45, S 5.62; found C 5.89, H 2.62, N 2.42, S 5.78. Anal. calcd for NdC_3_H_15_NO_15_P_3_S: C 6.27, H 2.63, N 2.44, S 5.58; found C 5.91, H 2.58, N 2.43, S 4.75. Anal. calcd for EuC_3_H_15_NO_15_P_3_S: C 6.19, H 2.60, N 2.41, S 5.51; found C 6.09, H 2.70, N 2.54, S 5.73. Anal. calcd for GdC_3_H_15_NO_15_P_3_S: C 6.13, H 2.57, N 2.38, S 5.46; found C 5.38, H 2.60, N 2.23, S 5.80. Anal. calcd for TbC_3_H_15_NO_15_P_3_S: C 6.12, H 2.57, N 2.38, S 5.44; found C 6.14, H 2.62, N 2.27, S 5.74.

Bimetallic solid solutions with nominal compositions [Ln(1)_1.6_Ln(2)_0.4_(H_4_NMP)_2_(H_2_O)_4_](HSO_4_)_2_·4H_2_O were isolated for the lanthanide combinations Eu_0.8_Tb_0.2_ and Tb_0.8_Eu_0.2_ using the same procedure described above. Anal. calcd for Eu_1.6_Tb_0.4_C_6_H_38_N_2_O_34_P_6_S_2_: C 5.82, H 3.09, N 2.26, S 5.17; found C 5.36, H 3.01, N 2.18, S 4.80. Anal. calcd for Tb_1.6_Eu_0.4_C_6_H_38_N_2_O_34_P_6_S_2_: C 5.78, H 3.07, N 2.25, S 5.14; found C 5.43, H 2.99, N 2.36, S 5.10.

### Structural Determinations

Single-crystal X-ray diffraction data for Pr^3+^, Nd^3+^, Sm^3+^, and Tb^3+^ derivatives of **Series I** were collected at room temperature with a Bruker D8 Venture using graphite-monochromated Mo Kα (λ = 0.71073 Å) radiation or a Rigaku AFC12 goniometer equipped with an enhanced sensitivity (HG) Saturn724+ detector mounted at the window of an RF-E+ Super Bright molybdenum rotate anode generator with HF Varimax optics (100 m focus, λ = 0.770 Å). The structures were solved by the direct methods,^37^ which revealed the position of all non-hydrogen atoms. These atoms were refined on F^2^ by a full-matrix least-squares procedure using anisotropic displacement parameters.^38^ Hydrogen atoms were placed at calculated positions and refined using a riding model, except for the water, HSO_4_^–^ anion, and phosphonic acid O–H hydrogens, which were located from the Fourier difference density maps and refined using a riding model with O–H distance restraints. The Olex2^39^ or SHELXTL software was used as a graphical interface.

The crystal structures of the remaining compounds of **Series I** (Ln^3+^ = Eu, Gd, Er, and Yb) were determined from synchrotron X-ray powder diffraction data (SXRPD) or laboratory X-ray powder diffraction data (LXRPD) using the Rietveld method^40^ and the crystal structure of the Tb^3+^ derivative as the starting model. SXRPD were collected at the high-resolution BL04-MSPD beamline of ALBA, the Spanish Synchrotron Radiation Facility (Barcelona, Spain). A wavelength of 0.4124 Å was selected with a double-crystal Si (111) monochromator and determined from a Si640d NIST standard (*a* = 5.43123 Å) measurements, using an MYTHEM detector. The capillary was rotated during data collection to improve diffracting particle statistics, and patterns were collected over the angular range of 0.33–44.5° (2θ). LXRPD for other compounds of both **Series I** and **II** were collected on a D8 ADVANCE (Bruker AXS) diffractometer equipped with a Johansson Ge(111) primary monochromator giving a monochromatic Mo radiation (λ = 0.7093 Å) and using the energy-dispersive linear detector LYNXEYE XE 500 μm. Data were collected in the angular region of 2.5–45° (2θ), with a step size of 0.01° and a counting time of ∼1536 s/step. For compounds **SD-Tb-I**, **Eu-I-230**, and **Tb-I-230**, the crystal structures were obtained by Rietveld refinement using as a starting model the structure of [La_2_(H_4_NMP)_2_(H_2_O)_3_(SO_4_)]·2H_2_O (CCDC# 1496873).^41^ For compounds of **Series II** (Ln^3+^ = Pr, Nd, Eu, Gd, and Tb), the crystal structure was solved for the Tb^3+^ derivative (**Tb-II**) by the direct methods using the program EXPO2014.^42^ Partial structural models were obtained, and the missing atoms were localized by difference of Fourier maps. All crystal structures were optimized by the Rietveld method^40^ using the program GSAS^43^ and the graphic interface EXPGUI.^44^ The following soft constraints were imposed in order to preserve chemically reasonable geometries for the phosphonate, amine, and sulfate groups. The soft constrains were /PO_3_C tetrahedron/P–O (1.53(1) Å), P–C (1.80(1) Å), O–O (2.55(2) Å), O–C (2.73(2) Å); /N(CH_2_)_3_ amine group/N–C (1.50(1) Å), C–C (2.45(2) Å), N–P (2.68(2) Å), and /SO_4_ tetrahedron/S–O (1.46(1) Å) and O–O (2.45(2) Å). No attempts to locate the H atoms were carried out due to the limited quality of the XRPD data. For the other compounds of this series, **Pr-II** and **Gd-II**, their structures were obtained by Rietveld refinement using the structure of **Tb-II** as the initial model.

Thermodiffractometric data for **Tb-I**, **Er-I**, and **Tb-II** were obtained for the samples loaded in an Anton Paar HTK1200N Camera, under static air, on a PANanalytical X’Pert Pro automated diffractometer. Data were collected in the Bragg–Brentano reflection configuration with Cu Kα_1_ and the X’Celerator detector. Data were collected at different temperature intervals from room temperature to 250 °C. A heating rate of 5 °C·min^–1^ and a delay time of 5 min to ensure thermal stabilization were used. XRPD patterns were recorded in the region of 4–70° (2θ) with a step size of 0.017–0.033° and an equivalent counting time of ∼57–100 s/step. A crystallographic study at 95% relative humidity (RH) and different temperatures was also collected for **Tb-I** on a D8 ADVANCE (Bruker AXS) using an Anton Paar MHC-trans chamber. Data were collected between 30 and 80 °C using a heating rate of 5 °C/min. The data range was between 3 and 20° (2θ) with a step size of 0.02° and an equivalent counting time of ∼384 s/step. Samples were held at each temperature for 10 min before recording any pattern, giving sufficient time for any transformation to take place.

### Particle Size Determination

The particle size measurements were carried out using a Zetasizer Nano ZS from Malvern. Particle suspensions were prepared at a concentration of 9 mg/mL in isopropanol, and they were stirred and sonicated in a water bath for six sequential cycles of 10 min to obtain a homogeneous dispersion. The size was determined via dynamic light scattering (DLS) using a 632.8 nm wavelength laser as the light source. Then, the measurement was performed measuring backscattered light at 173° in order to minimize multiple scattering, and the results were averaged and presented in intensity (raw data). The samples were measured in 1 cm path length disposable polystyrene cuvettes.

### Photoluminescence (PL) Studies

Photoluminescence spectra of powders were carried out on a Carl Zeiss Axio Imager.A2m microscope in fluorescence mode using an HBO 100 mercury lamp. The PL signal was collected using an Ocean Optics QE Pro spectrometer connected to the microscope via a fiber optic waveguide.

### Proton Conductivity Studies

Impedance measurements of the powdered polycrystalline compounds were carried out on cylindrical pellets (∼5 mm diameter; ∼0.9–1.1 mm thickness) obtained by pressing ∼30–40 mg of sample at 250 MPa for 1 min between porous C electrodes (Sigracet, GDL 10 BB, no Pt). Sample cells were placed inside a temperature- and humidity-controlled chamber (Espec SH-222), and impedance data were collected using an AUTOLAB PGSTAT302N or HP4284A impedance analyzer over the frequency range from 0.1 or 20 Hz to 1 MHz with an applied voltage of 0.35 or 1 V, respectively. Measurements were electronically controlled using the Nova or WinDETA package of programs.^45^ In order to equilibrate the water content, pellets were first preheated (0.2 °C/min) from 25 to 80 °C at 75 and 95% RH. Impedance spectra were recorded on cooling using stabilization times of 5 h for each temperature (80, 70, 60, 50, 40, 30, and 25 °C). Water condensation on the sample was avoided by reducing first the relative humidity before decreasing the temperature. The total pellet resistance (*R*_T_) was obtained from the intercept of the spike and/or the arc (low-frequency end) on the *Z*’ axis from the Nyquist plots. In addition, for the sulfate-deficient compounds (**SD-Ln-I**), samples were kept at 80 °C and 95% RH for 80–90 h, washed with DI water, and dried in air.

### Modeling Approaches

#### Ab Initio Electronic Structure Calculations

Plane wave DFT electronic structure calculations were performed for one unit cell of **Tb-I** using the Quantum Espresso package.^46−48^ The PBE functional and PAW pseudopotentials were used for all atoms with the exception of Tb, where a PAW + GGA pseudopotential was used. The 4f electrons of Tb were treated as valence electrons. Calculations were done using a 170 Ry kinetic energy cutoff for the wave function and 680 Ry for the kinetic energy cutoff for the charge density and potential. The convergence of energy with kinetic energy cutoff was investigated (Figure S1). A total of 450 bands were calculated. Only the Gamma point was used for all calculations. The valence charge density of the material was extracted, and using the chargemol code, the DDEC6 partial charges were calculated.^49,50^ Partial charge distribution for the material **Tb-I** can be found in Figure S2 and Table S1.

#### Diffusion Coefficient and Proton Conductivity

The first step in all calculations of proton conductivity is to compute the mean squared displacement, MSD, of the protons, as a function of simulation time, for an ensemble of N protons
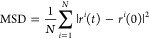
1

The diffusion coefficient *D* of a particle type is connected to the mean squared displacement MSD, and especially for free diffusion, their relationship is linear as shown
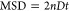
2

In this case, it is possible to calculate the diffusion coefficient from eq 3
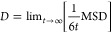
3and the conductivity using the Nernst–Einstein equation
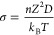
4

In this work, eq 3 will be used to extract the diffusion coefficient, and hence, the diffusion processes are treated as linear ones. In the simulations done in this work, the acidic hydrogens of the structure (hydrogen sulfate and phosphonic acidic hydrogens) were used as the source of diffusive protons. Calculation of the MSD for various temperatures resulted in calculations of σ as a function of *T*. Inserting log[σ] and 1000/*T* in an Arrhenius plot extracts the apparent activation energy for the proton conduction process (Figure S3).

#### Molecular Dynamics Simulations

The LAMMPS code was used for all classical MD simulations.^51^ A single unit cell was used for all simulations. The system was treated as an NVT ensemble, and a Nose–Hoover thermostat was used to control the temperature. Most of the bonding force field parameters were derived using ab initio techniques. The bonds and angles were treated as harmonic, and relative stretching and bending constants *k*_stretch_ and *k*_bending_ were calculated at the DFT/B3LYP/6-311G++(d,p) level. The bond and angle terms of the Tb–O bonds and O–Tb–O angles were taken from the Universal force field.^52^ The OPLS Lennard-Jones parameters^53−55^ were also used for all elements with two exceptions (Table S2). For terbium atoms, the UFF values were used, and for protons, the LJ parameters were optimized, so the OH bond length would be reproduced in the simulations. For the calculation of the force field parameters, initial structures of the fragments HSO_4_^–^, H_2_O, and H_4_NMP^2–^ were drawn with Avogadro software and were optimized at the UFF level.^56^ The UFF geometries were further optimized at the DFT/B3LYP/6-311G++(2d,2p) level for all fragments using the Gaussian code, G03 version.^57^ After the DFT optimization, the Hessian matrix was calculated to find the second derivatives of the energy with respect to the geometry. Using the Seminario method implemented in the VFFDT code^58^ and the Hessian matrix of each fragment, the bonding and angle force field parameters were derived. In order to keep the modeled crystal structure as stable as possible, the bond lengths and equilibrium angles were constrained to the values of the original structure. The acidic protons of the structure (hydrogen sulfate and phosphonate acidic hydrogens) were treated as nonbonded particles in the molecular dynamics simulations. The partial charges that were assigned to all atoms in the simulations are the DDEC6 charges. A summary of the process used for the calculations of the bonding parameters is shown in Figure S4.

Using purely ab initio charges, it was found that protons practically did not diffuse. Hence, the conductivity was heavily underestimated. The reason for that was considered to be the strength of electrostatic forces or limitations associated with fixed charge or point particle simulations that do not include atomic charge polarization models. So, as a charge scaling approach, the dielectric constant was varied, and short runs were executed to find an optimum value, so protons diffused at lower temperatures, and this value was found to be ε = 2ε_ο_ (Figure S5). The short simulations, for the investigation of the best dielectric constant value, were run for 1 ns each, with a 0.1 fs time step, at 50 °C. The longer 5 ns runs were done using a time step of 0.5 fs. A global cutoff of 10.0 Å was applied for both Lennard-Jones and close-range electrostatic interactions in all simulations. Long-range electrostatic interactions were treated using the Ewald summation scheme implemented in LAMMPS. The protons were grouped so that the MSD of the group could be calculated. To accelerate and observe diffusion of protons, high-temperature MD runs needed to be done. In order to extract the temperature dependence of the diffusion coefficient, the temperature was varied from run to run with simulations conducted at 35, 100, and 150 °C. Modeling the system as an NVT ensemble constrained the structure to the crystallographic one, so no phase transitions were observed during the high-temperature simulations. Lower-temperature properties were extrapolated from the high-temperature data using the Arrhenius and the Nernst–Einstein equations.

From the MSD data, the conductivity was calculated using the following form of the Nernst–Einstein equation
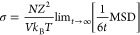
5The low-temperature data were calculated using the Arrhenius form of the conductivity
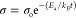
6Using *Z* = 1 as the charge of protons, *N* = 16 as the number of acidic protons, *V* as the volume of the unit cell, the proton conductivity values from the MSD and activation energy were derived (Tables S3–S4).

### Preparation of Composite Membranes and Characterization

Composite membranes, **N/SD-Eu-I** and **N/Tb-II**, containing 3% of each solid were prepared. As a representative example, the preparation process of the **N/Tb-II** composite membrane is described in detail. 18 mg of **Tb-II** was dispersed in 2-propanol (3 mL) via magnetic stirring (10 min) and sonication (10 min), in six subsequent cycles. The suspension was slowly added to the 20 wt % Nafion solution (3 g) in a flask under magnetic stirring for ∼2 h, giving rise a viscous homogeneous suspension. The membrane was prepared by casting using a blade (BYK Instruments) onto a polycarbonate plate that was allowed to air-dry for 48 h. Finally, the obtained membrane (with a thickness of 35–40 μm) was peeled off from the polycarbonate plate. The pristine Nafion membrane was also prepared using the same procedure. All membranes were characterized by XRPD, thermal analysis under N_2_, SEM (FEI, Helios Nanolab 650), and energy-dispersive X-ray spectrometry (X-Max Oxford). Water uptake of the composite membranes was determined by measuring the weight variation before and after hydration. The samples, three specimens for each membrane, were first dried under vacuum at 60 °C for 24 h, and the weights were measured. Then, the samples were immersed in deionized water for 24 h, wiped with tissue paper to remove any excess water on the film surface, and rapidly weighed. A stable wet weight was remained after 48 h in distillated water. The water uptake was calculated as a weight gain (wt %) referred to the dried sample.

### Membrane Electrode Assembly (MEA) Preparation

For both the anode and cathode, the catalyst layer ink consisted of Pt/C (40% Pt on Vulcan XC-72, E-TEK) with a Pt load of 1 mg Pt·cm^–2^, 30% Nafion (1100 EW), and 2:1 isopropanol/water mixture as the dispersion medium. After sonicating for 3 h, the catalyst ink was sprayed onto a nonwoven carbon paper gas diffusion layer with a Microporous Layer (MPL) and 5% PTFE-treated (SIGRACET 39 BC GDL) using an automatic spraying system EFD.

### Electrochemical Characterization of MEAs and In Situ Through-Plane Proton Conductivity

An experimental 5 cm^2^ single cell (ElectroChem Inc.) was used as cell hardware for all the electrochemical measurements. Performance tests were carried out using a Scribner 850e multi range fuel cell test system using hydrogen and oxygen (200 mL·min^–1^). The experiments were conducted under atmospheric pressure at 70, 80, and 90 °C cell temperatures and at 100% relative humidity of the gases. Polarization and power density curves were acquired only after the cell reached stable conditions, i.e., potential remained constant over time at a fixed current. Through-plane proton conductivities of the membranes were determined on the MEAs at 70, 80, and 90 °C and 100% RH by means of electrochemical impedance spectroscopy (EIS) using a potentiostat Autolab PGStat30 equipped with an FRA module. The cell was continuously supplied (200 mL^.^min^–1^) with humidified hydrogen (SHE, anode) and nitrogen (cathode). The amplitude of the sinusoidal signal was 10 mV, and the frequency range was 100 kHz to 10 Hz. The spectra were recorded under a DC bias potential of 0.45 V. The through-plane proton conductivities of the membranes σ_TP_ (S^.^cm^–1^) were obtained using the following equation: σ_TP_ = *L*/*RS*, where *L* is the membrane thickness (cm) and *S* is the active area of the MEA (5 cm^2^). Durability tests were performed at 80 °C and 100% relative humidity by maintaining the operating MEA at a constant voltage of 0.5 V for 28,000 s. The supplied current density was recorded during the extent of the experiment.

## Results and Discussion

The two polymorph series [Ln_2_(H_4_NMP)_2_(H_2_O)_4_](HSO_4_)_2_·*n*H_2_O (Ln = Pr^3+^, Nd^3+^, Sm^3+^, Eu^3+^, Gd^3+^, Tb^3+^, Er^3+^, and Yb^3+^ and *n* = 4–5; **Series I**) and [Ln(H_5_NMP)]SO_4_·2H_2_O (Ln = Pr^3+^, Nd^3+^, Eu^3+^, Gd^3+^, and Tb^3+^; **Series II**) were isolated by slow crystallization at 25 °C. Polymorphs of **Series I** were obtained by varying the molar ratio Ln^3+^/H_6_NMP/H_2_SO_4_/H_2_O from 1:5:19.3:3715 up to 1:5:135:3715, while those of **Series II** required addition of higher H_2_SO_4_ (95%) amounts up to a ratio 1:5:193:3715. Appropriate ratio adjustments were made depending on the lanthanide ion. These synthetic strategies resulted in the formation of mixed-ligand complexes in solution as precursors to the crystalline product phases. In addition, for **Series I**, bimetallic solid solutions can be prepared within a wide range of compositions, i.e., Eu_0.8_Tb_0.2_ and Tb_0.8_Eu_0.2_. Photoluminescence properties of **Tb-I**, **Sm-I**, and bimetallic solids were measured (Figures S6 and S7). In all cases, the emission that is recorded for the lanthanide ion containing samples arises from direct excitation of the lanthanide ion. The H_6_NMP and SO_4_^2–^ ligands play no detectable role in the emission of light.^33^ However, in the spectrum of **Eu_0.8_Tb_0.2_-I**, the most intense peak corresponds to the Tb^3+ 5^D_4_ → ^7^F_5_ transition, which demonstrates the greater relative intensity of the emission from Tb^3+^ than from Eu^3+^ (Figure S7), whatever the molar percentage of both lanthanide ions was. This was evidenced further in the overlapping peak around 620 nm (Eu^3+ 5^D_0_ → ^7^F_2_ and Tb^3+ 5^D_4_ → ^7^F_3_ transitions), where the compositional change to much greater Eu^3+^ content does not have an appreciable effect upon the relative intensity of the aggregated peak with respect to the peak at 542 nm (Tb^3+ 5^D_4_ → ^7^F_5_ transition). There is, however, a more pronounced splitting of the composite peak, which differentiates the Eu^3+^ and Tb^3+^ contributions, which arises from the increased contribution of the Eu^3+^ emission to this line.

### Structural and Stability Features of **Series I** and **II**

The crystal structures of **Series I** compounds were solved by single-crystal X-ray diffraction or Rietveld analysis (Figure S8) for the other members of the series. These solids crystallize in the monoclinic system, with space group *P*2_1_/*c*, with all atoms situated in general positions (Table 1 and Table S1). The basic layered crystal structure, illustrated for the Tb^3+^ derivative, resembles that found for [Ln(H_4_NMP)(H_2_O)_2_](Cl)·2H_2_O,^33^ in which the metal ion is surrounded by two coordinated water molecules and six oxygen atoms from four different H_4_NMP^2–^ ligands. Each zwitterionic H_4_NMP^2–^ ligand links four Ln^3+^ ions. Two H_4_NMP^2–^ ligands are bidentate, forming eight-membered rings, and the remaining two are monodentate, resulting in isolated LnO_8_ polyhedra (Figure S9).

**Table 1:** Crystallographic Data for Some Representative Members of **Series I** and **II**

**phase**	**Tb-I**	Pr-I*	**SD-Tb-I**	**Tb-I-230**	**Tb-II**
space group	*P*2_1_/*c*	*P*2_1_/*c*	*P*2_1_/*c*	*P*2_1_/*c*	*P*-1
chemical formula	C_6_H_38_N_2_O_34_P_6_S_2_Tb_2_	C_6_H_38_N_2_O_34_P_6_S_2_Pr_2_	C_6_H_34_N_2_O_29_P_6_STb_2_	C_6_H_22_N_2_O_26_P_6_S_2_Tb_2_	C_3_H_15_NO_15_P_3_STb
formula mass (g·mol^–1^)	1250.18	1214.14	1134.09	1106.06	589.06
λ (Å)	0.71073	0.71073	0.4132	0.7093	0.7093
*a* (Å)	8.4314(2)	8.6510(4)	8.5720(2)	8.8138(3)	10.0792(5)
*b* (Å)	19.2357(5)	19.5394(9)	16.2769(5)	16.0812(7)	9.5779(4)
*c* (Å)	10.5627(3)	10.7660(5)	10.5651(2)	10.3891(3)	9.3920(4)
α (°)	90.0		90.0	90.0	110.696(3)
β (°)	109.373(3)	109.922(2)	109.6499(11)	109.9755(19)	111.583(3)
γ (°)	90.0		90.0	90.0	101.511(4)
*V* (Å^3^)	1616.10(8)	1710.93(14)	1388.26(7)	1383.92(11)	729.91(6)
*Z*	2	2	2	2	2
temperature (K)	150.0(2)	100	298	298	298
range data (°)	2.71–30.62	2.09–27.55	2.00–42.49	4.20–47.98	4.18–48.00
independent reflections	3244	3943	7847	2183	2297
data/restrains/ parameters	3715/0/237	3943/52/321	6814/68/131	4428/51/122	4430/50/121
*R*_WP_			0.0682	0.0921	0.1109
*R*_P_			0.0479	0.0684	0.0835
*R*_F_			0.0188	0.0250	0.0393
*R* factor [*I* > 2σ(*I*)]	*R*_1_ = 0.0302^a^; *wR*_2_ = 0.0572^b^	*R*_1_ = 0.0334^a^; *wR*_2_ = 0.0994^b^			
*R* factor (all data)	*R*_1_ = 0.0386^a^; *wR*_2_ = 0.0597^b^	*R*_1_ = 0.0373^a^; *wR*_2_ = 0.1018^b^			
GoF	1.035	1.096			
CCDC number^c^	1980608	2003911	1980609	1980604	1980607

a *R*_1_(*F*) = Σ∥*F*_o_| – |*F*_c_∥/Σ|*F*_o_|.

b *wR*_2_(*F*^2^) = [Σ*w*(*F*_o_^2^ – *F*_c_^2^)^2^/Σ*F*^4^]^1/2^.

c CCDC contains the supplementary crystallographic data for this work.

**Figure 1:**
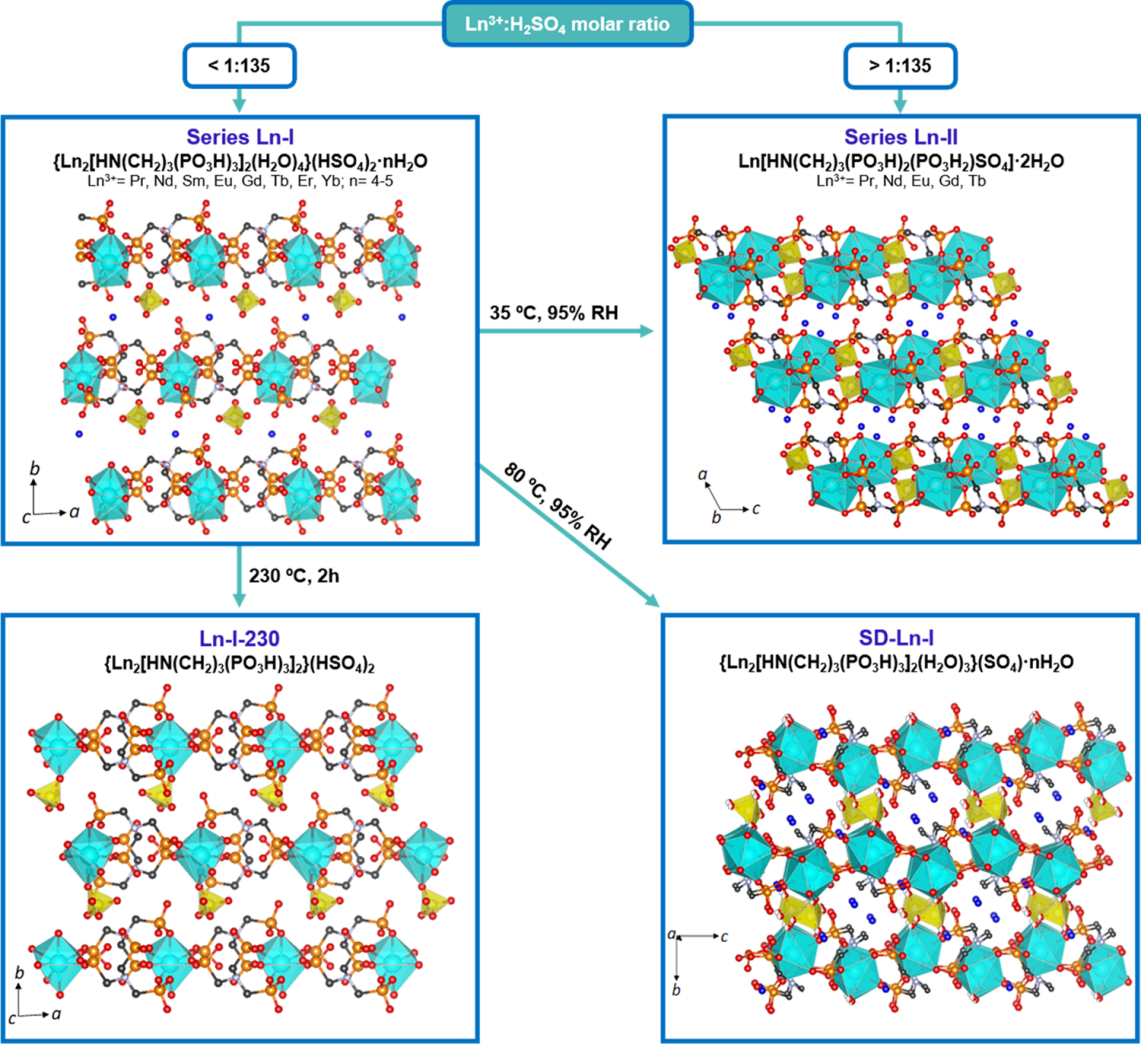
Phase transformations of Ln^3+^-nitrilotris(methylenephosphonates). Yellow tetrahedra represent hydrogen sulfate/sulfate groups, blue polyhedra represent LnO*_n_*, and lattice water molecules are shown as blue spheres.

Single-crystal data indicate that sulfate is present in the structure as a HSO_4_^–^ ion, as three S–O bond lengths are found between 1.466(3) and 1.451(3) Å, whereas the fourth one is much longer at 1.537(3) Å, as observed in the structure of Tb(SO_4_)(HSO_4_).^59^ Moreover, crystallographic sites are fully occupied, which rules out the presence of SO_4_^2–^ ions. The noncoordinated HSO_4_^–^ ions, together with lattice water molecules, are located between the cationic inorganic–organic layers (Figure 1). Importantly, the HSO_4_^–^ anions form hydrogen bonds through all four oxygen atoms with the coordinated waters (Ow1 and Ow2), lattice waters (Ow3 and Ow4), the protonated phosphonate oxygens (−P–O–H, O2, O5, and O8), and with the deprotonated phosphonate groups (O3, O6, and O9). These interactions create extended unidirectional H-bonds along the *a*-axis (Figure S10, Table S6).

A structural deviation in **Series I** was found for the Pr^3+^ derivative upon slightly modifying the amount of H_2_SO_4_ (95%) added to obtain a Pr^3+^/H_6_NMP/H_2_SO_4_/H_2_O molar ratio of 1:5:95:3000. These synthesis conditions are the same as those used by Mendes and Almeida Paz^41^ albeit without a microwave treatment. The crystal structure of this compound, {Pr_2_[(H_4_NMP)_2_(H_2_O)_4_](HSO_4_)_2_·4H_2_O}, designated as Pr-I*, was solved from single-crystal data and reveals the existence of two sulfate groups, each one having a half-site occupancy. Its structure features a PrO_8_ coordination environment in which one of the two coordinated water molecules is now half-substituted by a monodentate HSO_4_^–^ ion, coordinated through O11B (Figure S11a). The remaining uncoordinated HSO_4_^–^ ion is located, together with two lattice water molecules, in the interlayer region. As for the structure of the other **Series I** compounds, the layers are built up from PrO_8_ polyhedra interconnected via the phosphonate moieties. The sulfate ions interact with both coordinated and lattice water molecules and the oxygen atoms from the phosphonate groups, forming extended unidirectional hydrogen bonds (Figure S11b and Table S7). Compounds [La_2_(H_4_NMP)_2_(H_2_O)_3_(SO_4_)]·*n*H_2_O (*n* = 2–8) previously reported^41^ can be envisioned as structural analogues of **Series I** compounds because they present similar layered features (*a* and *c* parameters) but with variable interlayer distances (*b* axis) in order to accommodate different contents and arrangements of lattice water and SO_4_^2–^ ions.

**Figure 2:**
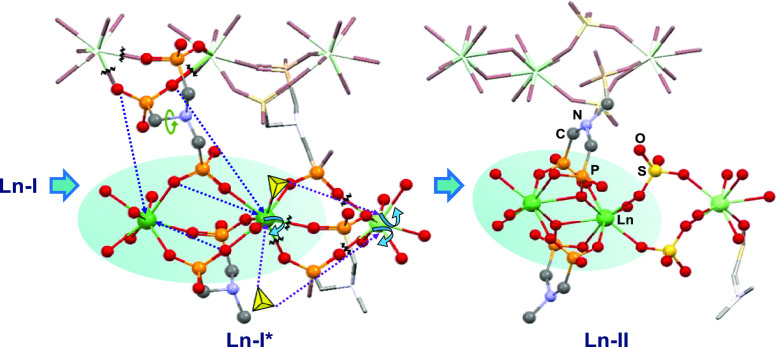
Tentative mechanism of dimer formation upon **Ln-I** to **Ln-II** transformation. Yellow tetrahedra represent hydrogen sulfate/sulfate groups, and bent arrows represent leaving coordinated water.

The crystal structures of compounds of **Series II**, with general formula [Ln(H_5_NMP)](SO_4_)]·2H_2_O (Ln^3+^ = Pr, Nd, Eu, Gd, Tb), were solved by X-ray powder diffraction data (Figure S12). These solids crystallize in a triclinic unit cell (Table 1 and Table S5) with all atoms located in general positions. Their structures consist of chains of dimeric Ln_2_O_14_ polyhedra connected by bridging sulfate ions running along the *c-*axis (Figures 1 and 2). These chains are linked together through the phosphonate groups in the *bc*-plane (Figure S13). The Ln_2_O_14_ dimers, containing eight-coordinated lanthanide centers, are formed by bridging via O1 and O2 belonging to one phosphonate (P1). In addition, another phosphonate (P2) acts simultaneously as a chelate (O4 and O6) for each Ln^3+^ ion while bridging (via O6) the two Ln^3+^ ions of the dimer. The third phosphonate (P3) binds exclusively in a monodentate fashion to the Ln^3+^ ions (Figure S13). Furthermore, the structure contains two lattice water molecules situated and forming H-bonding interactions between the layers (Figure S10). Extended bidimensional (*bc* plane) H-bonding networks (Table S8) result from linkages mainly between lattice water (Ow1 and Ow2), the phosphonate oxygen atoms (O7 and O4), and the sulfate oxygen atom (Os2). This conduction pathway, therefore, contrasts with that found for the compounds of **Series I**, which is preferentially unidirectional.

The stability of both families of compounds was studied by thermal analysis and thermodiffraction. Solids of **Series I** progressively lose water upon heating, accompanied by structural changes before decomposition over 300 °C (Figure S14). The thermodiffraction patterns indicate that the solids exhibit a phase transformation at ∼100–120 °C before complete dehydration, accompanied by a significant weight loss, as detected in the TG curves. The crystalline phase that remains at 230 °C is consistent with an anhydrous structure (i.e., for **Tb-I**, the observed weight loss accounts for 8 water molecules per formula 11.8%, calculated 11.5%). The heavier Ln^3+^ derivatives, with a slightly higher water content, follow a similar behavior, except that the appearance of partially dehydrated intermediate phases starts at markedly lower temperatures (60 °C for Er^3+^) before full dehydration (observed weight loss for 9 water molecules 14.3%, calculated 12.6%). Thus, a different weight loss profile is observed in the corresponding TG curves (Figure S14). The compounds remain stable up to 250–270 °C before ligand decomposition occurs.

The anhydrous compounds of **Series I** crystallize in a monoclinic cell (Table 1 and Table S5 and Figure S15) with parameters similar to those of the precursor phase, preserving the same connectivity between the ligand phosphonate groups and the seven-coordinated Ln^3+^ ions within the layer (Figure S16). The Ln^3+^ coordination environment contains one oxygen atom originating from the sulfate group instead of two coordinated water molecules, as noticed for the **Series I** compounds. The sulfate group also interacts through hydrogen bonding with oxygen atoms (O1, O3, and O6) from the phosphonate groups from the adjacent layer (Table S9).

Compounds of **Series I** experience an irreversible solid-state transformation to phase **II** at 35 °C and 95% RH (Figure 1) as confirmed by powder diffraction. This transformation implies the transfer of a proton from a HSO_4_^–^ group to one of the phosphonate ligands. The process is thought to be water-mediated because this transformation is not observed upon dry heating of the solid up to 230 °C (Figure S14). As a result, the metal–ligand connectivity changes from 1:4 to 1:3, while the sulfate group substitutes the coordinated water molecules and bridges the newly formed Ln_2_O_14_ dimers (Figure 2). The isolated compound Pr-I*, with the HSO_4_^–^ ions coordinated in a monodentate fashion to the lanthanide ion, can be considered as an intermediate stage during this transformation.

On the other hand, samples of **Ln-I** solids, which were studied by impedance spectroscopy, i.e., were heated at 80 °C and 95% RH, converted into a phase formulated as [Ln_2_(H_4_NMP)_2_(H_2_O)_3_](SO_4_)·*n*H_2_O, (Ln = Pr, Nd, Eu, Gd, Tb and *n* = 4–5); hereinafter **SD-Ln-I** (Figure 1). According to elemental analyses and PXRD patterns (Figure S17), the latter composition matches with that reported for the La^3+^ derivative [La_2_(H_4_NMP)_2_(SO_4_)]·*n*H_2_O (*n* = 2–11).^41^ This transformation results from partial dissolution of the initial compounds, as revealed by the presence of dissolved Ln^3+^ and phosphonate upon soaking the samples in water, which gives rise to solids having a sulfate content reduced by half. Solids **SD-Ln-**I remain stable for, at least, 4 days at 80 °C and 95% RH, as demonstrated by XRPD and thermal analysis. Interestingly, loss of sulfate was complete for the Yb^3+^ derivative. A similar behavior was previously observed for the isoreticular compounds [Ln(H_4_NMP)(H_2_O)_2_](Cl)·2H_2_O.^33,35^

The crystal structure of the sulfate-deficient derivative, **SD-Ln-I**, has been reported elsewhere,^41^ and here, we report the crystallographic data for Tb^3+^ derivative (**SD-Tb-I**), from Rietveld refinement (Table 1, Figure S18). For **SD-Ln-I** compounds containing other Ln^3+^ ions, the limited quality of their XRPD patterns prevented further structural refinement. Formation of **SD-Tb-I** does not imply a change in charge and connectivity of the ligand, but simply a substitution of two uncoordinated HSO_4_^–^ ions and one coordinated water molecules by one SO_4_^2–^ ion coordinated in a monodentate fashion, through O10 (Figure 1). The SO_4_^2–^ ion points toward the interlayer region interacting via H-bonds with the noncoordinated oxygen atoms O3 and O6 from the phosphonate groups as well as with the coordinated water Ow1 and the lattice water Ow3 (Table S10). An extended H-bonding network is thus created along the *a*-axis, through interconnected lattice water Ow2 (Figure S10), which differs from that in **Series I** compounds.

In contrast to **Series I** compounds, those of **Series II** exhibit a unique low-temperature weight loss corresponding to removal of lattice waters, i.e., for **Tb-II**, observed 7.0%, calculated 6.0%, (Figure S19a). According to the thermodiffraction patterns (Figure S19b), solids remain crystalline up to 140 °C. Compounds progressively lose crystallinity above 140 °C until their ultimate thermal decomposition at ∼250 °C. The dehydration of these solids led to highly hygroscopic materials at high relative humidity values. The solids of this series remain stable in composition at high relative humidity (95%) and 80 °C (Figure S20).

### Proton Conductivity

The notion of incorporating anions to balance the cationic lanthanide-H*_x_*NMP layers^33,35^ opens possibilities for design of novel structures, but now containing proton conductivity-enhancing anions through the formation of extended H-bonding networks. Hence, the incorporation of sulfate ions serves this purpose and gives the opportunity to evaluate how these could influence proton conductivity.

**Figure 3:**
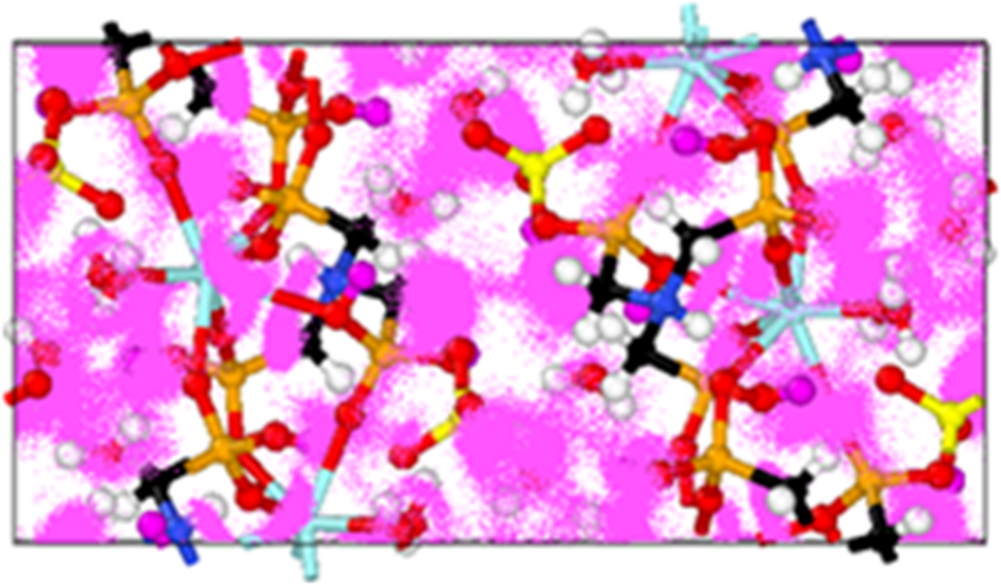
Proton trajectories occurring in a single unit cell.

Due to the instability of compounds of **Series I** in high humidity conditions, electrochemical impedance spectroscopy (EIS) measurements were conducted on **SD-Ln-I** and 230 °C-heated samples. Nevertheless, in an effort to understand the process of proton conduction in [Ln_2_(H_4_NMP)_2_(H_2_O)_4_](HSO_4_)_2_·*n*H_2_O, ab initio electronic structure calculations and classical molecular dynamics simulations were performed for **Tb-I**. Molecular dynamics (MD) simulations^51^ show that the proton conduction mechanism is Grotthuss-type (calculated activation energy 0.35 eV). The MD trajectories of the protons (Figure 3) show that lattice water molecules act as strong proton carriers since a large protonic density can be visualized around them. Coordinated phosphonate oxygens, as well as noncoordinated oxygens, also contribute. Nevertheless, from single-crystal X-ray diffraction data, there seem to be specific H-bonding pathways along the *a*-axis (Figure S10). The estimated proton conductivity values calculated from MD simulations range between 1.4 × 10^–3^ S·cm^–1^, at 35 °C, and 6.8 × 10^–3^ S·cm^–1^ at 80 °C.

**Figure 4:**
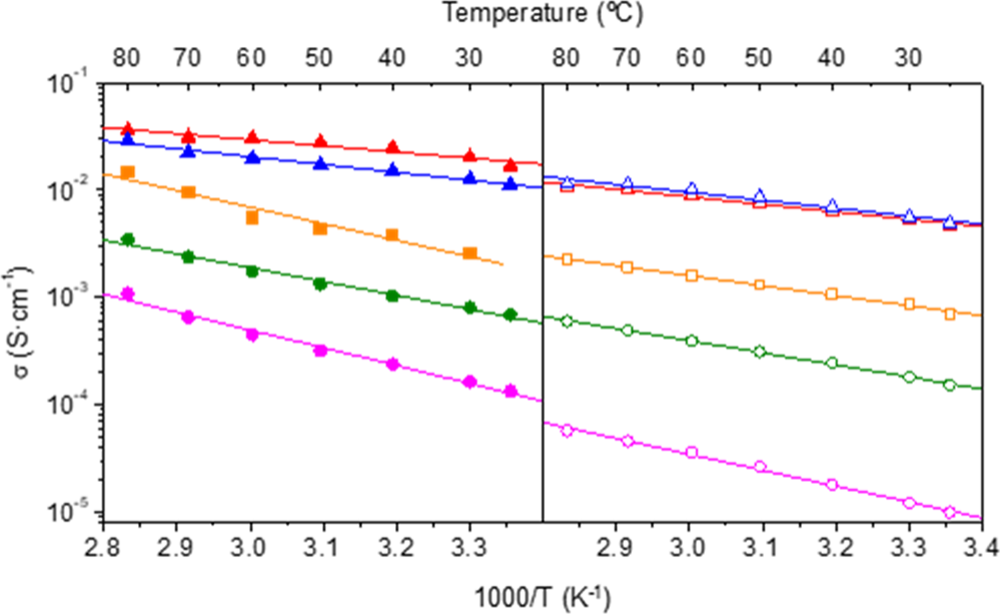
Arrhenius plots at 95% (closed symbols) and 75% RH (open symbols) for selected compounds of **Series I** and **II**: **SD-Tb-I** (yellow square), **Eu-I-230** (red triangle), **Tb-I-230** (blue triangle), **Pr-II** (green circle), and **Tb-II** (pink circle).

The proton conductivities obtained for **Ln-I** derivatives, from the Nyquist plots (Figures S21 and S22), are given in Table 2. All these compounds display high proton conductivities, ranging from ∼2.8 × 10^–3^ (Yb^3+^) to ∼3.6 × 10^–2^ S·cm^–1^ (Eu^3+^) at 80 °C and 95% RH, and activation energy values (0.11–0.31 eV) typical of a Grotthuss-type mechanism of proton transfer (Figure 4). Post-impedance analysis revealed that samples heated at 230 °C also show high water content, with weight loss between 8.2 (Tb^3+^) and 13.9% (Yb^3+^), up to 230 °C (Figure S23), as well as a tendency to evolve to sulfate-deficient materials according to the elemental analysis. However, their XRD patterns only show the presence of the initial 230 °C-heated phase with lower crystallinity, which would indicate that the adsorbed water is external to the crystalline phase.

**Table 2:** Proton Conductivity and Activation Energy Values for the Compounds of **Series I** and **II**

	**95% RH**		**75% RH**	
**sample**	**σ** (S·cm^–1^)	***E*_a_ (eV)**	**σ** (S·cm^–1^)	***E*_a_ (eV)**
SD-Tb-I	1.5 × 10^–2^	0.31	2.2 × 10^–3^	0.18
Eu-I-230	3.6 × 10^–2^	0.11	1.1 × 10^–2^	0.14
Tb-I-230	2.9 × 10^–2^	0.14	1.1 × 10^–2^	0.17
Yb-I-230	2.8 × 10^–3^	0.17	1.9 × 10^–4^	0.26
Pr-II	3.5 × 10^–3^	0.26	6.0 × 10^–4^	0.22
Tb-II	1.1 × 10^–3^	0.33	5.7 × 10^–5^	0.29

The proton conductivities of these materials decay at lower RH, a feature common in water-mediated proton conductors.^60^ In addition, these proton conductivity values are considerably higher than those of the analogous chloride-containing compounds [Ln(H_2_NMP)(H_2_O)_2_](Cl)·2H_2_O,^33^ indicating a prominent role of the sulfate ion in promoting efficient proton transfer. This enhancement of proton conductivity may be attributed to the high propensity of sulfate to establish H-bonding interactions with available species, such as water molecules and phosphonate groups. In addition, the obtained values are compared with those observed for Nafion 117 membranes^61,62^ and are among the highest values reported for phosphonate-based lanthanide CPs.^16,30,60,63−65^

Compounds of **Series II** exhibit systematically lower proton conductivity values than those of **Series I**, approximately 1 order of magnitude (Table 2). This is even more evident at lower relative humidity (75%). However, the proton conduction mechanism remains unchanged, as revealed by their low activation energy values. This behavior can be ascribed to the higher availability of sulfate oxygens present in compounds of **Series I** for establishing H-bonding networks, as shown by MD simulations. In light of this, it is important to note that the sulfate group in the structures of **Series II** compounds acts as a bridging group between two adjacent Ln^3+^ ions; hence, fewer sulfate oxygen atoms are available for H-bonding interactions. On the other hand, both **Series I** and **II** have similar morphological features, demonstrated by aggregates of elongated platy particles (Figure S24). However, from the SEM images, **Series II** particles are apparently smaller than those of **Series I**. Smaller particles are thought to decrease proton conductivity, as observed for other compounds.^30,65,66^

### Nafion-Mixed Membranes

**Figure 5:**
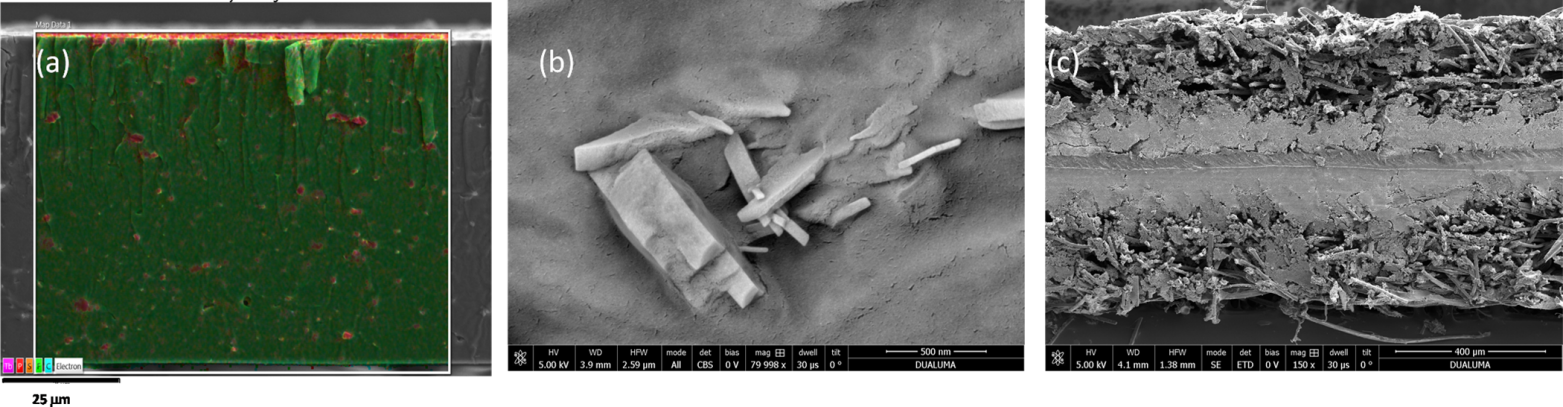
SEM images for **N/Tb-II** membrane: (a) cross-sectional SEM–EDX, (b) surface FE-SEM, and (c) cross-section of MEA after conducting the fuel cell durability test.

Two compounds showing high proton conductivity capabilities (one from each series, namely, **SD-Eu-I** and **Tb-II**) were selected for the preparation of composite membranes using Nafion as the supporting polymeric matrix and a load of the corresponding lanthanide phosphonate of 3% w/w (**N/SD-Eu-I** and **N/Tb-II**). This percentage was previously shown as the optimal composition.^30^ The powder diffraction patterns of all composite membranes display the characteristic diffraction peaks of the different crystalline lanthanide phosphonates together with the broad peak, between 12 and 20° (2θ), typical of the pristine Nafion membrane (Figure S25). In addition, the SEM micrographs of the composite membranes in combination with EDX analysis (Figure 5a and Figure S26), confirm a uniform distribution of the metal phosphonate particles inside the polymeric matrix, especially for **N/Tb-II**. On a nanometer scale, the surface FE-SEM images (Figure 5b and Figure S26) display the platy morphology of lanthanide phosphonate particles forming small aggregates fairly well integrated into the Nafion matrix.

Composite membranes adsorb 14–16% less water than pristine Nafion (Table 3), which may be attributed to the existence of a strong interaction between lanthanide phosphonate particles and the hydrophilic sulfonic groups of the organic polymer, thus limiting access of water molecules to these groups, as well as the filler particles.^67^ In fact, the sulfonate group binds to Ln^3+^ in lanthanide sulfophosphonates^30^ and is chemically analogous to the strongly Ln^3+^-binding sulfate ligands. Taking into account that a high water uptake may reduce the mechanical properties of a membrane, its decrease in these mixed membranes make them suitable for fuel cell applications.

**Table 3:** Proton Conductivities, Particle Sizes, and Water Uptake of Pristine Nafion and Nafion-Mixed Membranes

	**proton conductivity** (mS·cm^–1^)			**particle size**	
**composite membrane**	**70 °C**	**80 °C**	**90 °C**	***D*v(50)**	**water uptake**
Nafion	34.9	34.9	34.4		43.2
**N/SD-Eu-I**	31.9	34.4	31.4	1000	26.5
**N/Tb-II**	35.4	36.1	34.3	1370	29.5

**Figure 6:**
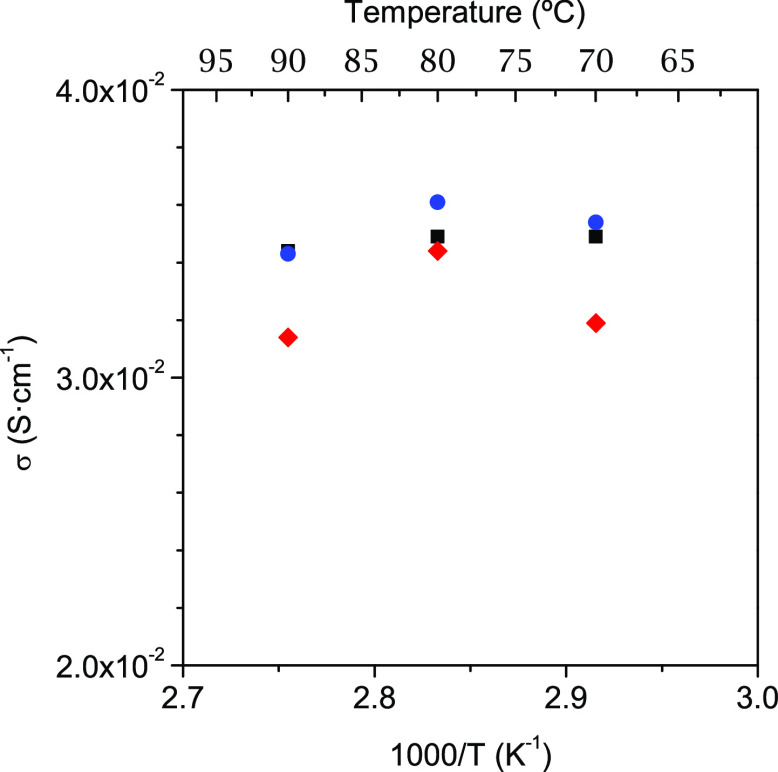
Proton conductivities, at 100% RH, for the pristine Nafion membrane (black square), **N/SD-Eu-I** (red diamonds), and **N/Tb-II** (blue circles) composite membranes.

Nafion-mixed membranes contain metal phosphonate particles with distribution particle sizes, *D*v(50) between 1000 nm, **SD-Eu-I**, and 1370 nm for **Tb-II** (Figure S27). In situ through plane proton conductivity values are similar for all membranes (Figure 6, Table 3), although slightly higher conductivity was found for **N/Tb-II**, likely due to its higher dispersion degree into the membrane and final particle size (Figure S27). However, all composite membranes show a small decay of the proton conductivity at 90 °C due, likely, to the loss of the optimal hydration degree of the membranes (Figure 6).

**Figure 7:**
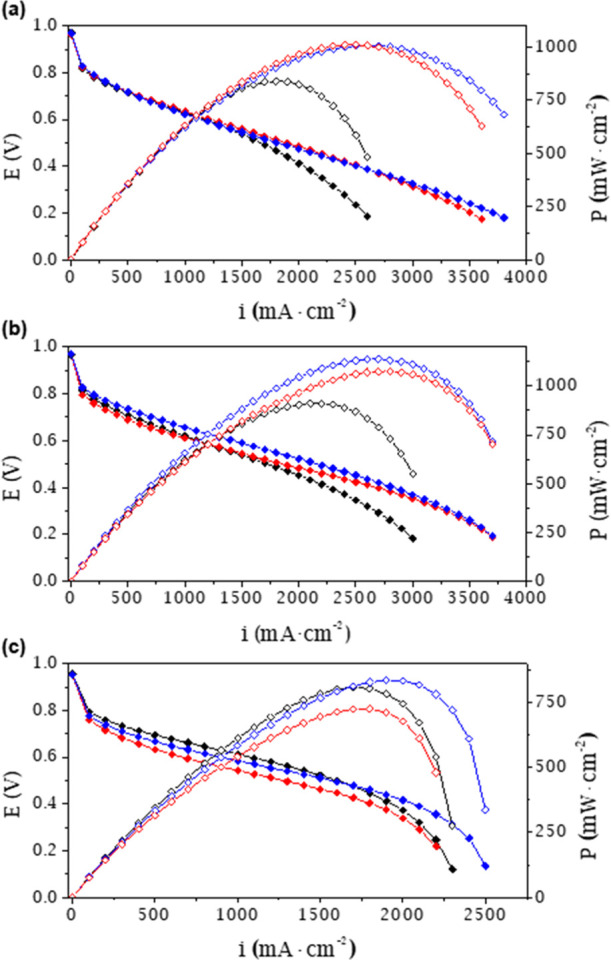
Polarization (full) and power density (empty) curves for pristine Nafion (black), **N/SD-Eu-I** (red), and **N/Tb-II** (blue) composite membranes at 100% RH and different temperatures of (a) 70, (b) 80, and (c) 90 °C.

In addition, all composite membranes were evaluated, by performance analysis in H_2_/O_2_ single cells, as proton exchange membranes for the PEMFC. Figure 7 shows the polarization and power density curves of the **N/SD-Eu-I** and **N/Tb-II** composite membranes as well as those of the pristine Nafion membrane, as a commercial reference material. The incorporation of **SD-Eu-I** or **Tb-II** into the Nafion polymeric matrix leads to composite membranes that perform satisfactorily in PEMFCs. All composite membranes show improved electrochemical features compared to the pristine Nafion membrane (Figure 7, Table 4), especially at 70 °C, reaching maximum current densities of 3601 and 3800 mA·cm^–2^ and maximum power densities of 1012 and 1008 mW·cm^–2^, for **N/SD-Eu-I** and **N/Tb-II**, respectively. At 90 °C, **N/SD-Eu-I** shows similar behavior to the pristine Nafion membrane, while the composite **N/Tb-II** membrane gives the best results of maximum current and power densities in the entire range of the measured temperatures (Table 4). The power and current density values obtained for these composite membranes are higher than those obtained for pristine Nafion (Table 4) and, to the best of our knowledge, represent the highest values reported for composite Nafion/MOF membranes.^68−70^

**Table 4:** Maximum Current and Power Density Values

	**max. current density** (mA·cm^–2^)			current density (0.5 V) (mA·cm^–2^)			**max. power density** (mW·cm^–2^)		
**membrane**	**70 °C**	**80 °C**	**90 °C**	**70 °C**	**80 °C**	**90 °C**	**70 °C**	**80 °C**	**90 °C**
Nafion	2600	3000	2299	1699	1700	1600	840	910	809
**N/SD-Eu-I**	3601	3700	2000	1899	1800	1299	1012	1075	725
**N/Tb-II**	3800	3700	2499	1800	2100	1600	1008	1138	836

**Figure 8:**
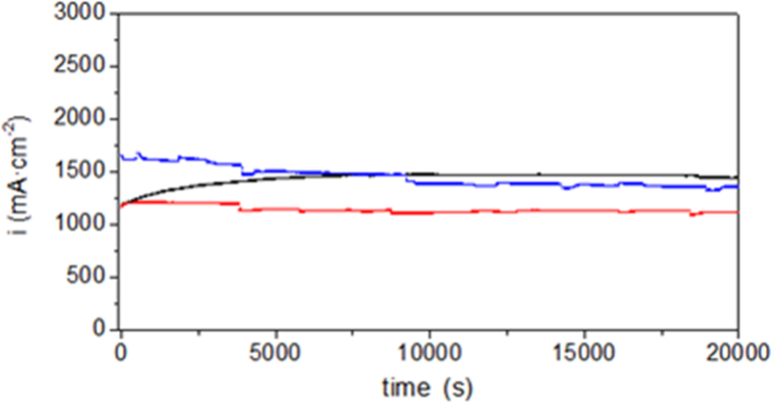
Current density curves through the time at constant voltage (0.5 V), 80 °C, and 100% RH for Nafion (black), **N/SD-Eu-I** (red), and **N/Tb-II** (blue).

Cell lifetime is an important factor to be considered for this particular application. Therefore, to complete the electrochemical characterization of the composite membranes, durability tests were performed maintaining the operating MEAs at constant voltage (0.5 V), 80 °C, and 100% RH during, approximately, 467 min. The current density curves recorded throughout the entire time are shown in Figure 8. The three MEAs perform efficiently along the entire experiment even though in the case of pristine Nafion a more stable behavior was observed from the beginning with a slight and continuous increase in the supplied current density up to around 300 min and then remaining practically constant afterward. At shorter times, the **N/Tb-II** membrane provides a somewhat higher current density than pristine Nafion although it gradually decreases over time and stabilizes posterior to 150 min. If the two composite membranes are compared, again **N/Tb-II** performs clearly better.

Once the electrochemical characterization was finished, the single cell was disassembled and the MEA was examined, verifying that its appearance was acceptable and did not present fractures, a fact that was corroborated by means of scanning electron microscopy of the cross sections (Figure 5c). Homogeneous and crack-free morphologies were observed, maintaining a good adhesion between layers so it can be concluded that the composite membranes are mechanically stable and durable under the operating conditions.

## Conclusions

In this work, we have examined the incorporation of sulfate ions into the layered structure of Ln^3+^-H*_x_*NMP coordination polymers in order to evaluate their performances as proton conducting fillers in PEMFCs. From a structural point of view, sulfate acts as a charge-compensating anion or plays a role as a bridging group in the mixed layers. For the former case, the polymorph series {Ln_2_[HN(CH_2_)_3_(PO_3_H)_3_]_2_(H_2_O)_4_}(HSO_4_)_2_·*n*H_2_O (Ln = Pr, Nd, Sm, Eu, Gd, Tb, Er, Yb; *n* = 4–5; **Series I**) was produced. A second series of materials (**Series II**) was obtained by increasing the concentration of sulfuric acid in solution, giving rise to the composition Ln[HN(CH_2_)_3_(PO_3_H_2_)(PO_3_H)_2_]SO_4_·2H_2_O (Ln = Pr, Nd, Eu, Gd, Tb). The latter solids also featured layers composed of edge-sharing dimeric Ln_2_O_14_ polyhedra, interconnected by the bridging sulfate ligands. In contrast to the compounds of **Series II**, heating the compounds of **Series I** led to complex dynamics of transformation at high relative humidity, yielding structures characteristic of **Series II**, at 35 °C, whereas sulfate-deficient networks was produced at 80 °C. All stable materials studied exhibited enhanced proton conductivity with respect to their Cl^–^-containing congeners, with values ranging from 1.1 × 10^–3^ to 3.6 × 10^–2^ S·cm^–1^ at 80 °C and 95% RH. Preliminary results indicate that these solids perform satisfactorily as fillers of Nafion-based membranes, showing power and current densities higher than those of the pristine Nafion in short times. Nevertheless, the time-dependent current density curves revealed, in the case of pristine Nafion, a slightly more stable behavior, likely due to its higher water uptake capacity.

## Supporting Information Available

Details of ab initio electronic structure calculations, molecular dynamics simulation, PL spectra, Rietveld plots, representative coordination environment of Ln ions of **Series I** and **II**, H-bond interactions, thermal analysis, XRPD patterns, thermodiffractometric studies, complex impedance plane plots, SEM–EDX images, force field parameters, computational results, crystallographic data, and H-bond distances (PDF)
Computed charge density (MP4)
