# Supplementary material for: Phase Transformation Dynamics in Sulfate-Loaded Lanthanide Triphosphonates. Proton Conductivity and Application as Fillers in PEMFCs
Source: ACS Appl Mater Interfaces. 2021 Mar 25;13(13):15279–91. doi: 10.1021/acsami.1c01441 (PMC8610370; doi:10.1021/acsami.1c01441)
Supplement: Supplementary file 1 — am1c01441_si_001.pdf [file am1c01441_si_001.pdf]

Supporting Information

For

# Phase Transformation Dynamics in Sulfate-loaded Lanthanide Triphosphonates. Proton Conductivity and Application as Fillers in PEMFCs

*Inés R. Salcedo,*[‡] *Rosario M. P. Colodrero,*[‡] *Montse Bazaga-García,*[‡] *M. López-González,*[§] *Carmen del Río,*[§] *Konstantinos Xanthopoulos,*[¥] *Konstantinos D. Demadis,*[¥] *Gary B. Hix,*[#] *Aleksandra D. Furasova,*[£] *Duane Choquesillo-Lazarte,*[⊥] *Pascual Olivera-Pastor*[‡,] ** and Aurelio Cabeza*[‡,] ***

[‡]Departamento de Química Inorgánica, Cristalografía y Mineralogía, Universidad de Málaga, Campus de Teatinos s/n, Málaga-29071, Spain.

[§]Instituto de Ciencia y Tecnología de Polímeros (CSIC), Juan de la Cierva 3, Madrid-28006, Spain.

[¥]Crystal Engineering, Growth and Design Laboratory, Department of Chemistry. University of Crete. Heraklion, Crete, GR-71003, Greece.

[#]School of Sciences, University of Wolverhampton, Wulfruna St, Wolverhampton, WV1 1LY, United Kingdom.

[£]Department of Physics and Engineering, ITMO University, St. Petersburg, 197101, Russia.

[⊥]Laboratorio de Estudios Cristalográficos, IACT (CSIC-UGR), Avda. de las Palmeras 4, 18100 Armilla, Granada, Spain.

Corresponding Author e-mail address:

* Prof. Dr. A. Cabeza, e-mail: aurelio@uma.es
* Prof. Dr. P. Olivera-Pastor, e-mail: poliverap@uma.es

## Table of contents:

### Figures:

**Figure S1.** (a) Convergence of energy with kinetic energy wave function cutoff; (b) Plotted density of states for **Tb-I**; and (c) Valence charge density computed at the PBE level rendered with VESTA.
**Figure S2.** DDEC6 partial charge distribution rendered with Jmol for **Tb-I**.
**Figure S3.** (a) MSD over time for several temperatures and (b) calculated Arrhenius plot for **Tb-I**.
**Figure S4.** Summary of the process used for the calculations of the bonding parameters and equations used for force field parameterisation.
**Figure S5.** MSD and log(σ) for various values of $\varepsilon_o$.
**Figure S6.** (a) PL spectra (λex= 355 nm) for **Tb-I** and (inset) its image under Hg lamp illumination; (b) PL spectra (λex= 355 nm) for **Sm-I**.
**Figure S7.** (a) PL spectra (λex= 355 nm) for **$Eu_{0.8}Tb_{0.2}$-I** and (b) PL spectra (λex= 355 nm) for **$Tb_{0.8}Eu_{0.2}$-I**. Black letters correspond to $Tb^{3+}$ transitions whereas in red letters are shown the transition assigned to $Eu^{3+}$.
**Figure S8.** Rietveld plots for (a) **Eu-I**, (b) **Gd-I**, (c) **Er-I** and (d) **Yb-I**.
**Figure S9.** (a) Representative coordination environment of $Ln^{3+}$ ions in **Series I** compounds and (b) detail of the metal-ligand connectivity in a single layer.
**Figure S10.** H-bond interactions for the representative compounds: (a) **Tb-I**, (b) **SD-Tb-I** and (c) **Tb-II** (lattices water in blue and coordinated water in green).
**Figure S11.** (a) Coordination environment and ligand connectivity for $Pr^{3+}$ ion for **Pr-I*** and (b) H-bond interactions along the *a*-axis.
**Figure S12.** Rietveld plots for (a) **Pr-II**, (b) **Gd-II** and (c) **Tb-II**.
**Figure S13.** (a) Representative coordination environment of $Ln^{3+}$ ions in **Series II** compounds and (b) detail of the connectivity of $Tb_2O_{14}$ dimers through the sulfate and phosphonates groups in a single layer.
**Figure S14.** (a) Thermal analysis for compounds **Nd-I** (purple), **Tb-I** (blue) and **Er-I** (magenta) and thermodiffraction patterns for (b) **Tb-I** and (c) **Er-I**.
**Figure S15.** Rietveld plots for (a) **Eu-I-230** and (b) **Tb-I-230**.
**Figure S16.** Coordination environment for **Eu-I-230**.
**Figure S17.** XRPD patterns for: (a) **SD-Pr-I** (olive) and **SD-Nd-I** (purple) compared with $[La_2(H_4NMP)_2(H_2O)_3(SO_4)]\cdot 6H_2O$ (black; CCDC no. 1496872); (b) **SD-Gd-I** (orange), **SD-Eu-I** (red) and **SD-Tb-I** (blue) compared with $[La_2(H_4NMP)_2(H_2O)_3(SO_4)]\cdot 2H_2O$ (black; CCDC no. 1496873).
**Figure S18.** Rietveld plot for **SD-Tb-I**.
**Figure S19.** (a) TG curves for **Pr-II** (olive), **Eu-II** (red) and **Tb-II** (blue); (b) thermodiffractometric study for **Tb-II**.
**Figure S20.** Thermodiffractometric studies at high relative humidity (95%) for: (a) **Tb-I** and (b) **Tb-II**.
**Figure S21.** Complex impedance plane plots for **SD-Tb-I** and **Ln-I-230** derivatives at 75 (left) or 95% RH (right) and different temperatures: 80 (black), 70 (red), 60 (green), 50 (blue), 40 (cyan), 30 (magenta) and 25 °C (yellow).
**Figure S22.** Complex impedance plane plots for **Series II** derivatives at 75 (left) or 95% RH (right) and different temperatures: 80 (black), 70 (red), 60 (green), 50 (blue), 40 (cyan), 30 (magenta) and 25 °C (yellow).
**Figure S23.** X-ray powder diffraction patterns (left) and thermal analysis (right) before (blue) and after (black) proton conductivity measurements for (a) **SD-Tb-I**, (b) **Tb-I-230** and (c) **Tb-II**.
**Figure S24.** SEM images of (a) **Eu-I**, (b) **SD-Eu-I** and (c) **Tb-II**.
**Figure S25.** XRPD patterns of (a) **SD-Eu-I** (green), **N/SD-Eu-I** composite membrane (olive) and Nafion® membrane (black) and (b) **Tb-II** (cyan), **N/Tb-II** composite membrane (blue) and Nafion® membrane (black).
**Figure S26.** Cross-section SEM-EDX (a) and surface FE-SEM (b) images for **N/SD-Eu-I**.
**Figure S27.** Particle size distribution for (a) **SD-Eu-I** and (b) **Tb-II**.

**Tables**:

**Table S1.** Elements and partial DDEC6 charges range used for the MD simulations.
**Table S2.** Force field parameters (Lennard Jones parameters) used in the simulations.
**Table S3.** Calculated proton conductivity values from the MSD.
**Table S4.** Extrapolated low temperature data using the Arrhenius equation for the conductivity in the range 20-80 $^{o}$C.
**Table S5.** Crystallographic data for compounds of **Series I** and **II.**
**Table S6.** H-bond distances for **Tb-I**.
**Table S7.** H-bond distances for **Pr-I***.
**Table S8.** H-bond distances for **Tb-II**.
**Table S9.** H-bond distances for **Tb-I-230**.
**Table S10.** H-bond distances for **SD-Tb**.

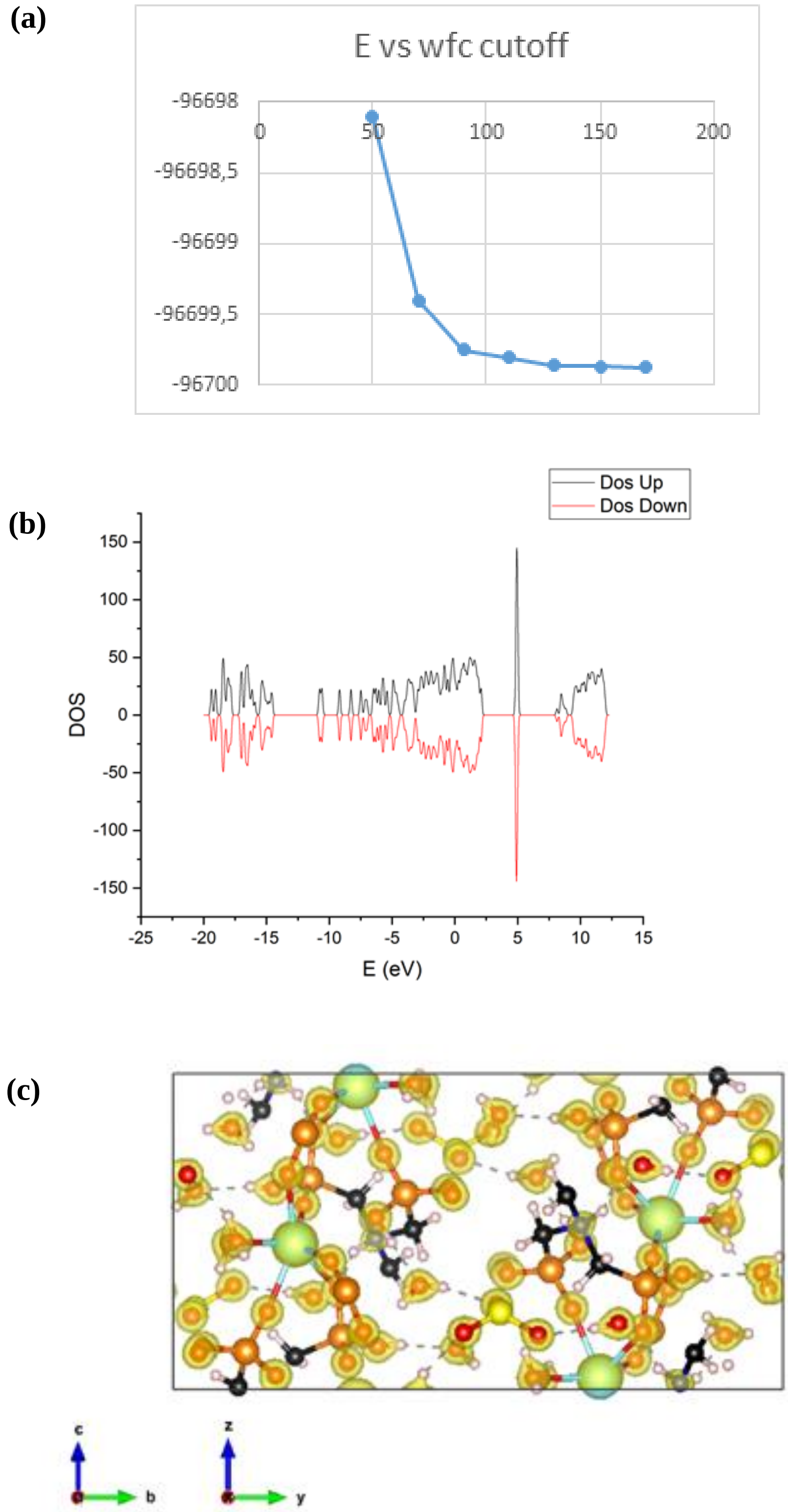


**Figure S1.** (a) Convergence of energy with kinetic energy wave function cutoff; (b) Plotted density of states for **Tb-I** (electronic band gab was calculated to be $E_{gap}$ = 2.5 eV and the fermi energy of the material $E_{Fermi}$ = 5.38 eV); and (c) Valence charge density computed at the PBE level rendered with VESTA.[1]

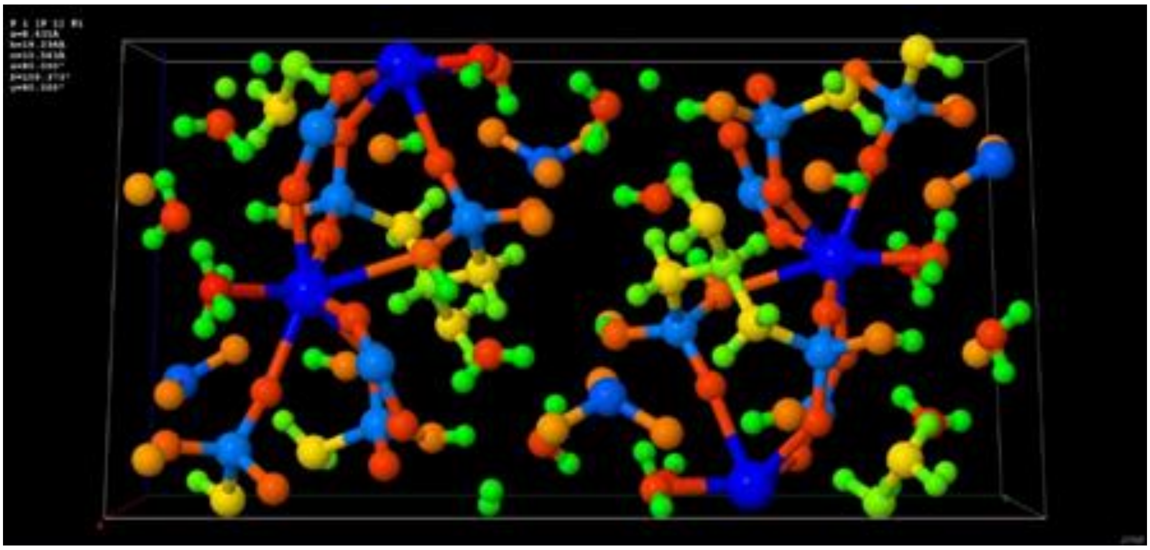

**Figure S2.** DDEC6 partial charge distribution rendered with Jmol for **Tb-I**.[2]

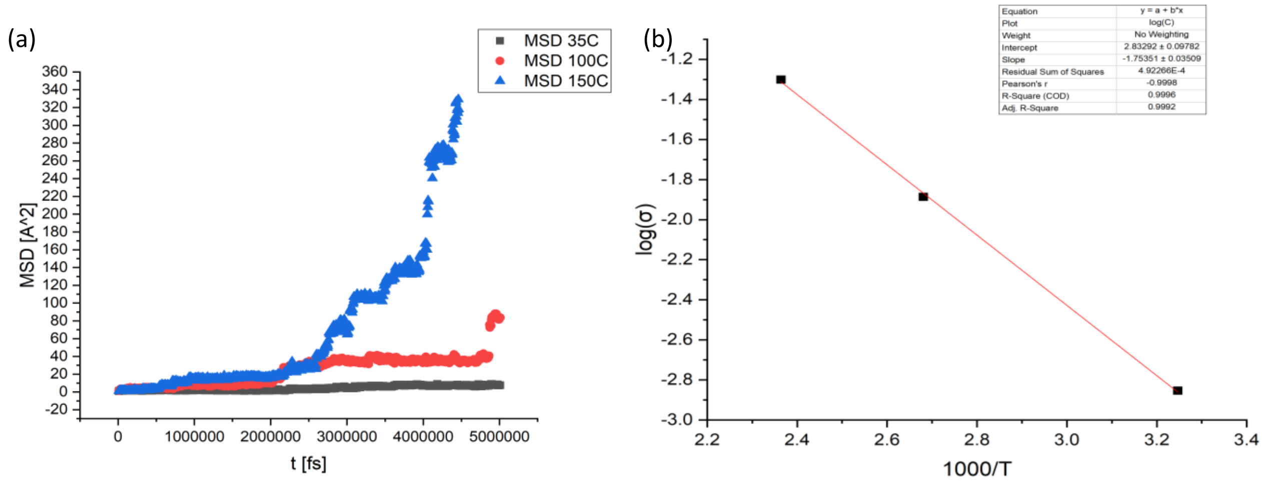


**Figure S3.** (a) MSD over time for several temperatures and (b) calculated Arrhenius plot for **Tb-I**.

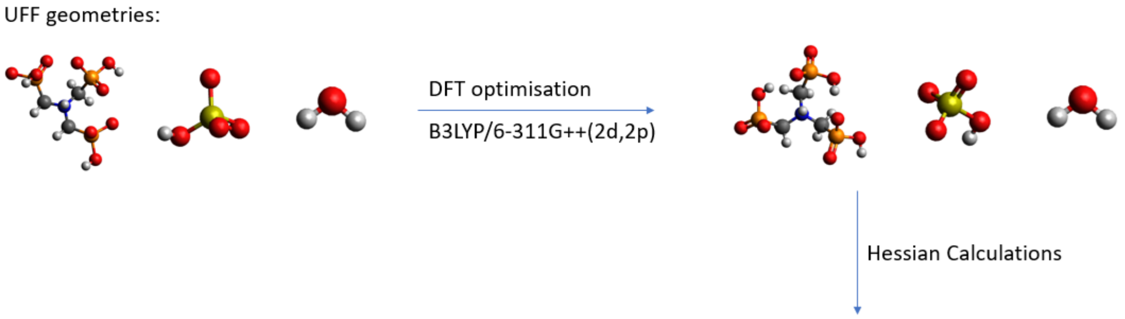


| Potential type | Equation | Source |
|---|---|---|
| Bonds | $E_{bond} = k_{bond}(r - r_0)^2$ | Seminario |
| Angles | $E_{angle} = k_{angle}(\theta - \theta_0)^2$ | Seminario |
| Dihedrals | $E_{dihedral} = k_{dihedral}[1 + d\cos(n\varphi)]$ | UFF |
| Lennard-Jones | $E_{LJ} = 4\varepsilon[\left(\frac{\sigma}{r}\right)^{12} - \left(\frac{\sigma}{r}\right)^{6}]$ | OPLS |
| Coulomb | $E_{\mathrm{Coulomb}} = \frac{1}{4\pi\varepsilon_o}\frac{q_1 q_2}{r^2}$ | DDEC6 |

Seminario method

Other source

$$H(E) = \begin{bmatrix} \frac{\partial^2 E}{\partial x_1^2} & \cdots & \frac{\partial^2 E}{\partial x_1 \partial x_n} \\ \vdots & \ddots & \vdots \\ \frac{\partial^2 E}{\partial x_n \partial x_1} & \cdots & \frac{\partial^2 E}{\partial x_n^2} \end{bmatrix}$$

**Figure S4.** Summary of the process used for the calculations of the bonding parameters and equations used for force field parameterisation.

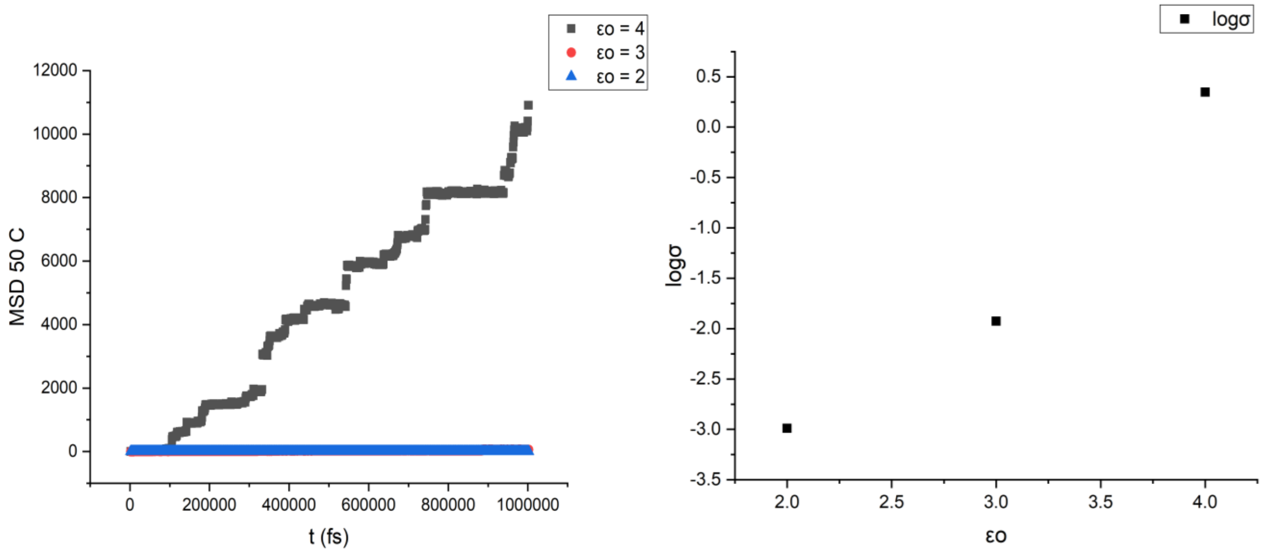


**Figure S5.** MSD and log(σ) for various values of $\varepsilon_o$.

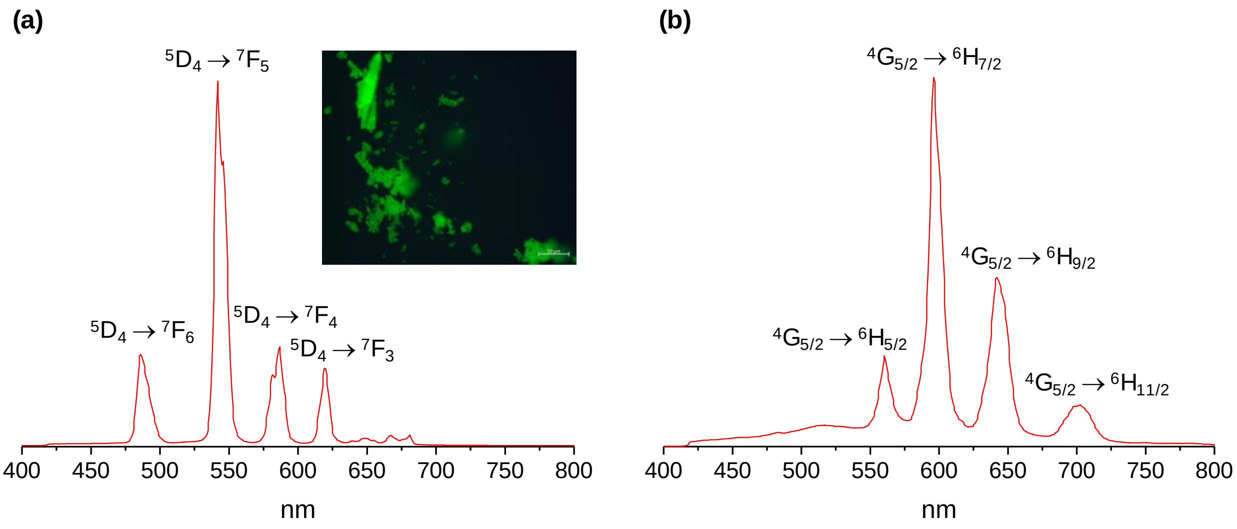


**Figure S6.** (a) PL spectra (λex= 355 nm) for **Tb-I** and (inset) its image under Hg lamp illumination; (b) PL spectra (λex= 355 nm) for **Sm-I**.

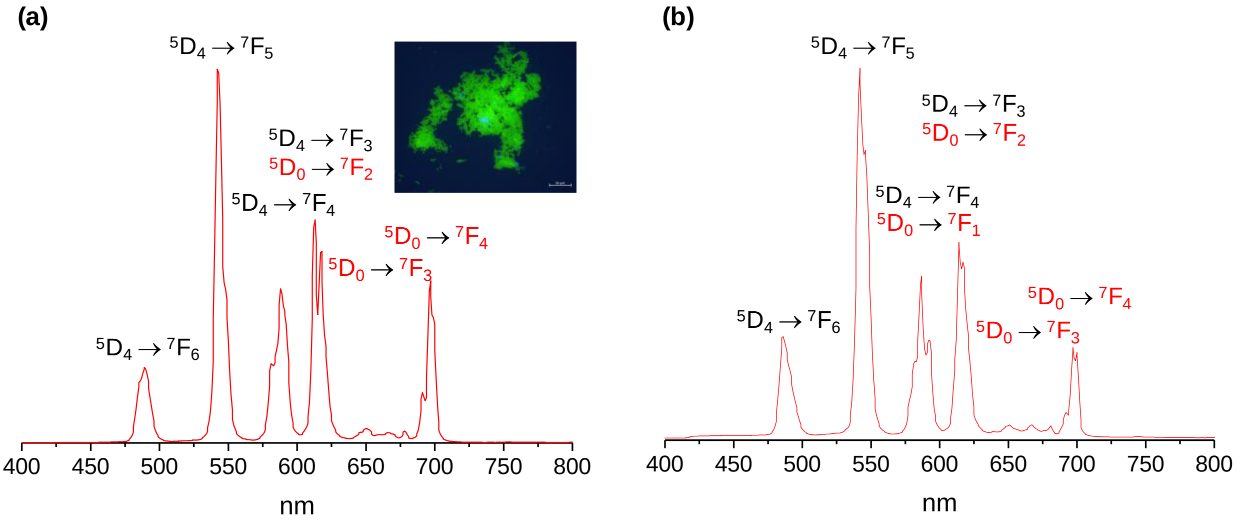


**Figure S7.** (a) PL spectra (λex= 355 nm) for **Eu$_{0.8}$Tb$_{0.2}$-I** and (b) PL spectra (λex= 355 nm) for **Tb$_{0.8}$Eu$_{0.2}$-I**. Black letters correspond to $Tb^{3+}$ transitions whereas in red letters are shown the transition assigned to $Eu^{3+}$.

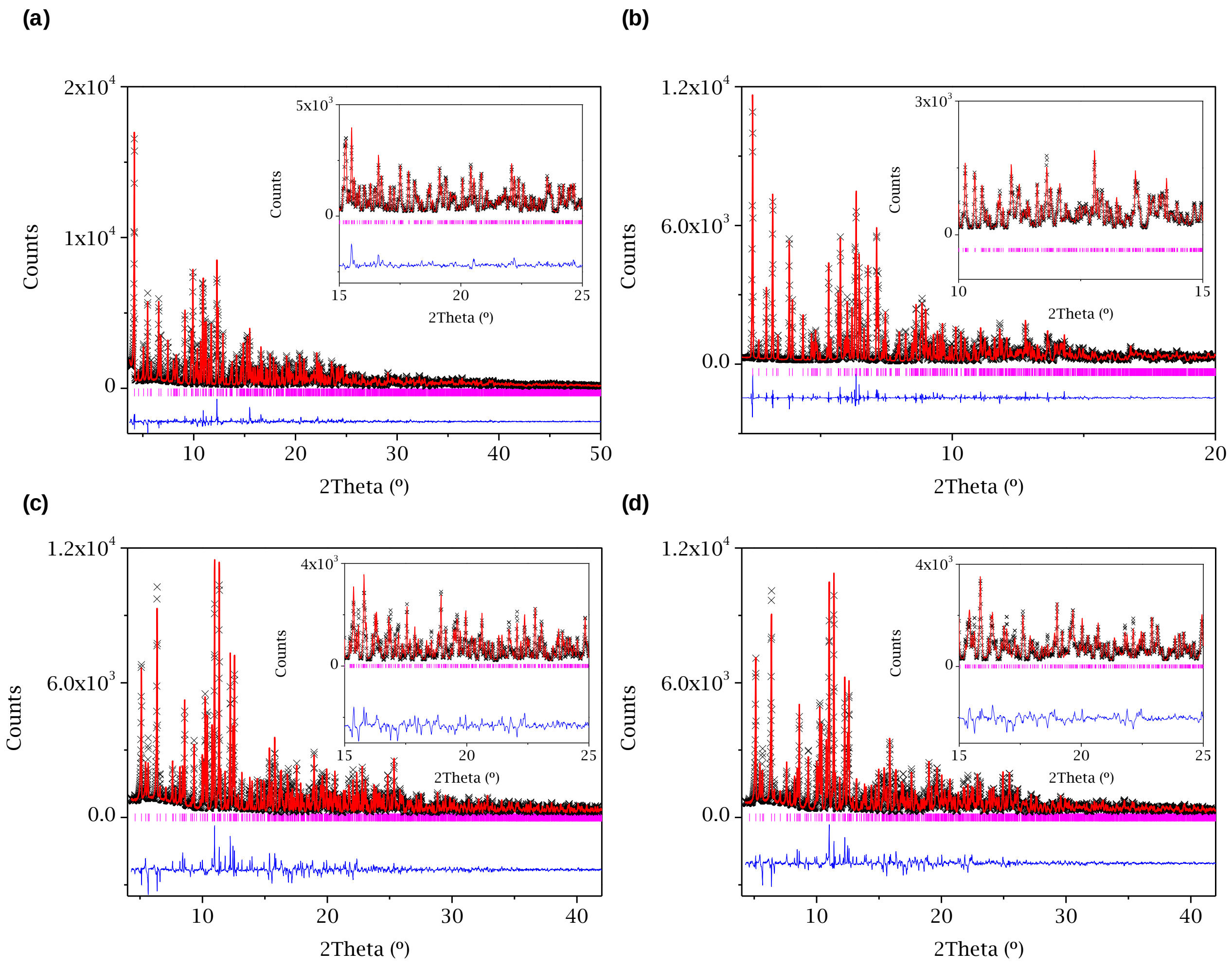


**Figure S8.** Rietveld plots for (a) **Eu-I**, (b) **Gd-I**, (c) **Er-I** and (d) **Yb-I**.

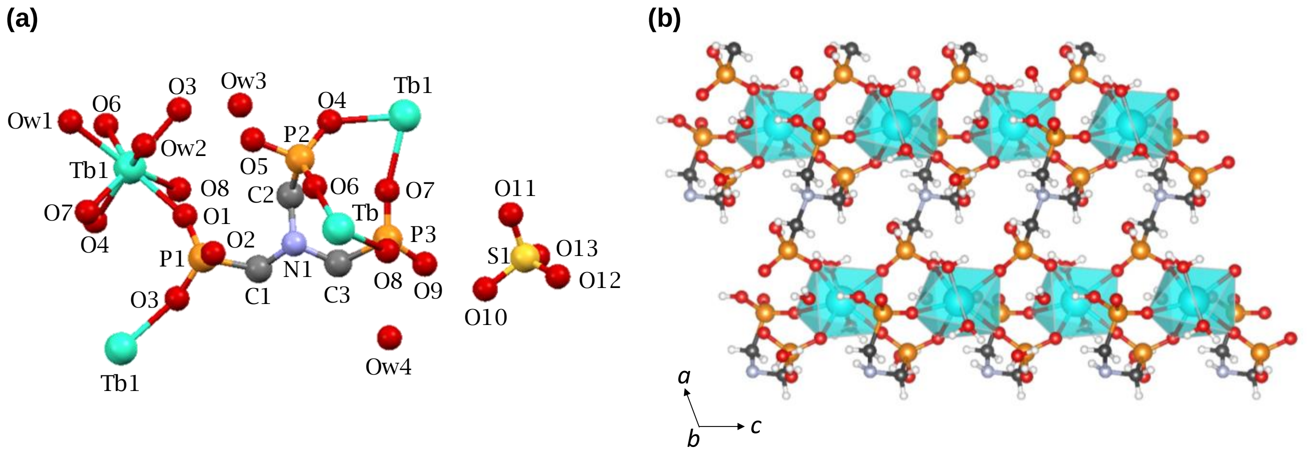


**Figure S9.** (a) Representative coordination environment of $Ln^{3+}$ ions in **Series I** compounds and (b) detail of the metal-ligand connectivity in a single layer.

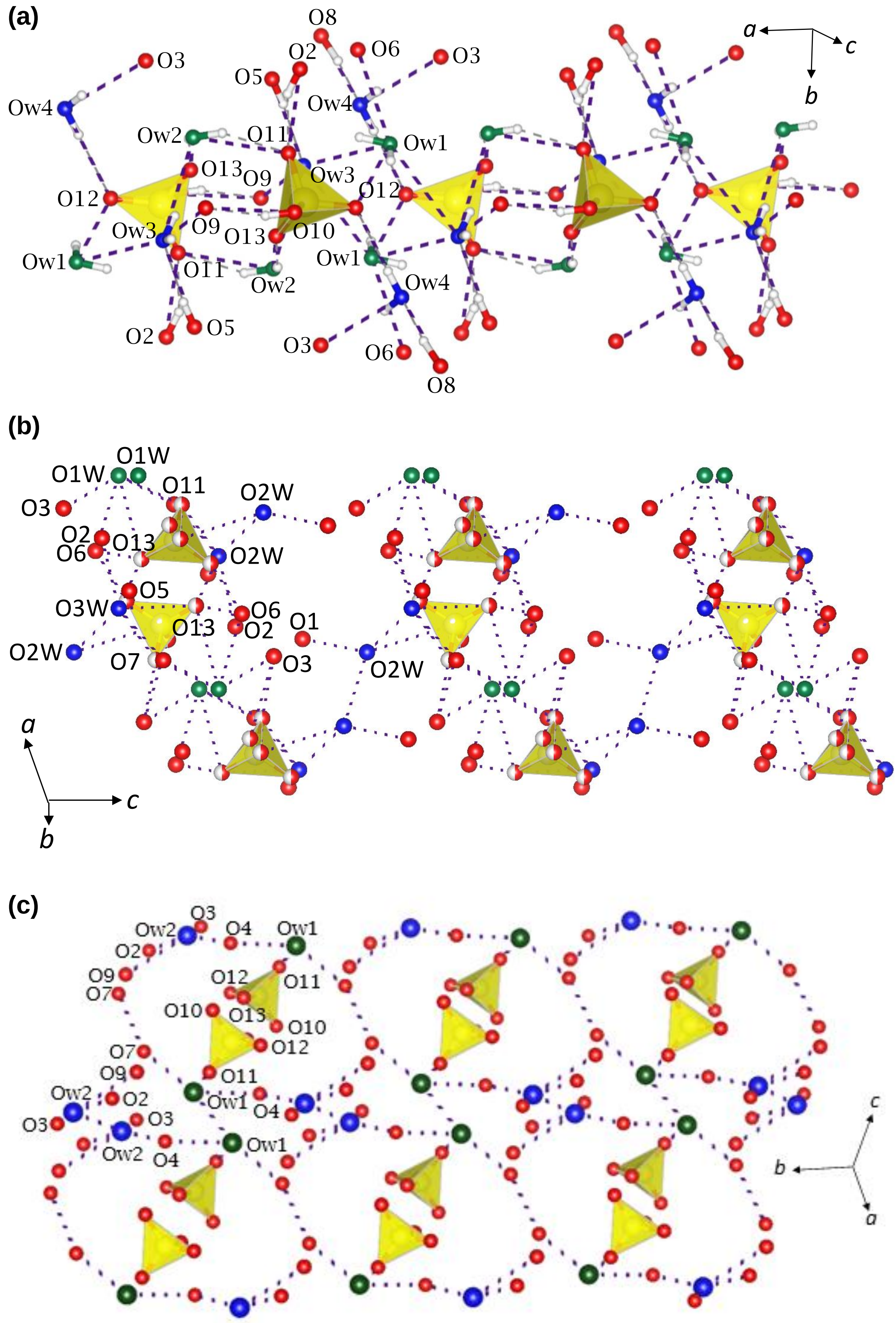


**Figure S10.** H-bond interactions for the representative compounds: (a) **Tb-I**, (b) **SD-Tb-I** and (c) **Tb-II** (lattices water in blue and coordinated water in green).

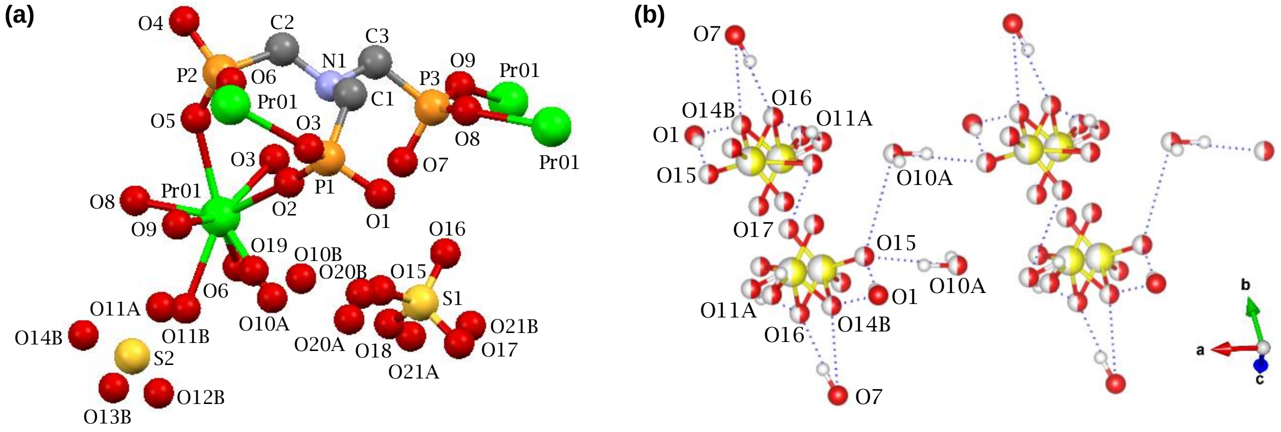


**Figure S11.** (a) Coordination environment and ligand connectivity for $Pr^{3+}$ ion for **Pr-I*** and (b) H-bond interactions along the *a*-axis.

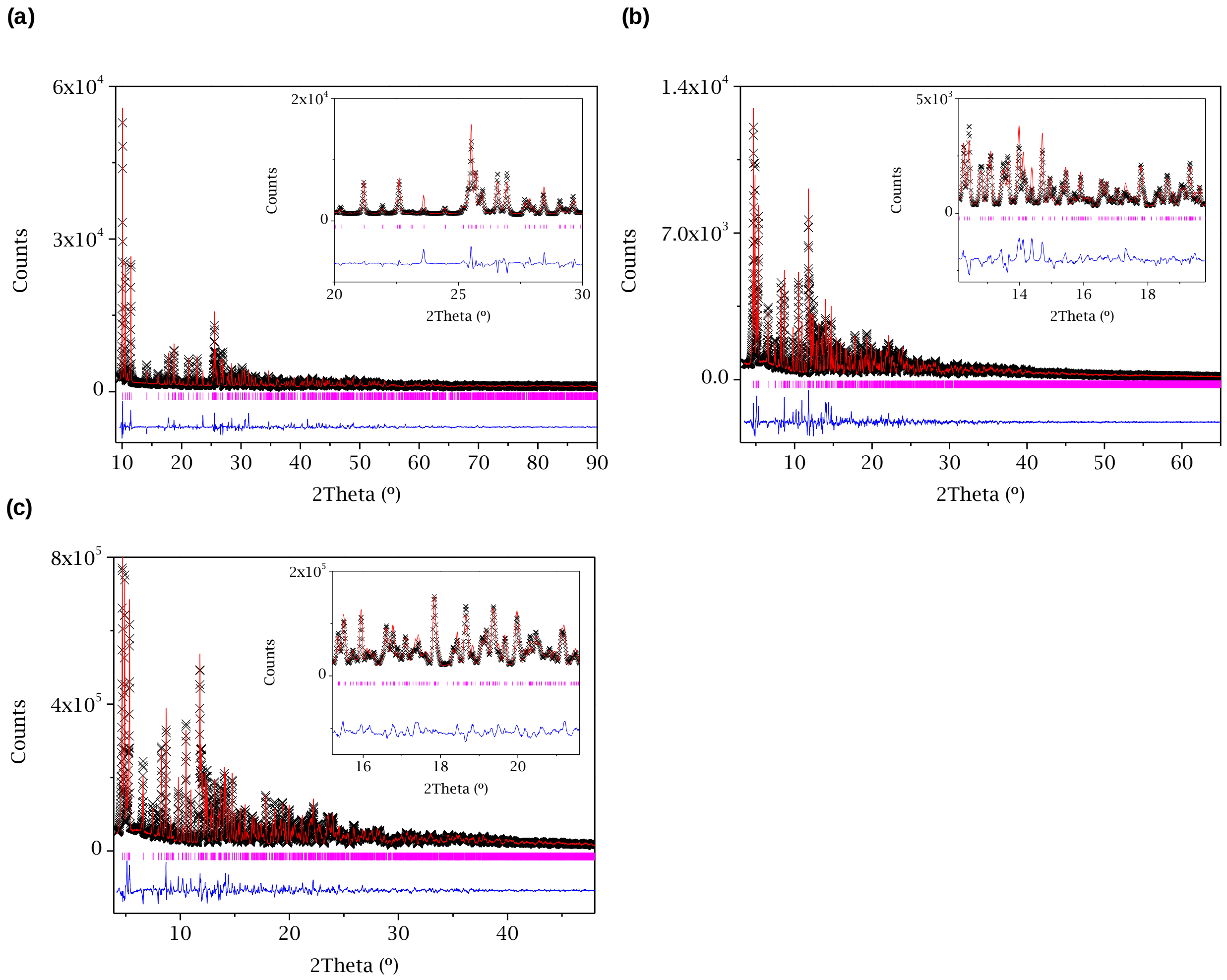


**Figure S12.** Rietveld plots for (a) **Pr-II**, (b) **Gd-II** and (c) **Tb-II**.

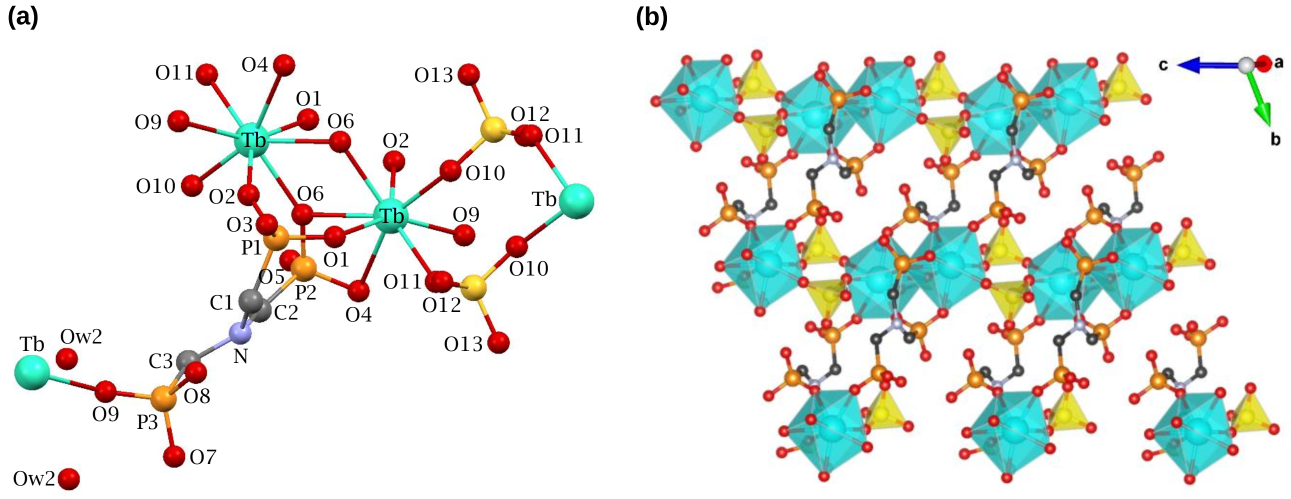


**Figure S13.** (a) Representative coordination environment of $Ln^{3+}$ ions in **Series II** compounds and (b) detail of the connectivity of $Tb_2O_{14}$ dimers through the sulfate and phosphonates groups in a single layer.

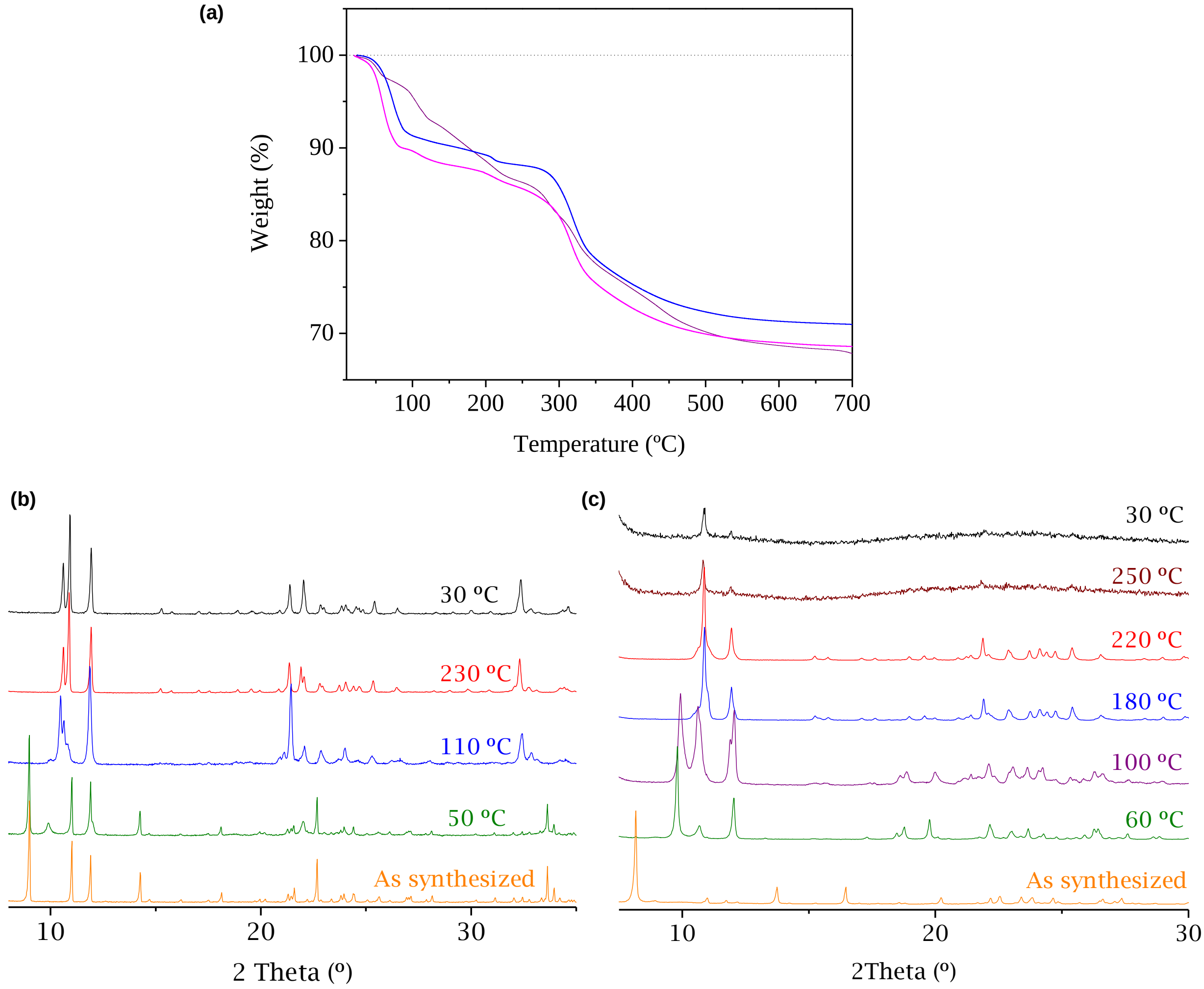


**Figure S14.** (a) Thermal analysis for compounds **Nd-I** (purple), **Tb-I** (blue) and **Er-I** (magenta) and thermodiffraction patterns for (b) **Tb-I** and (c) **Er-I**.

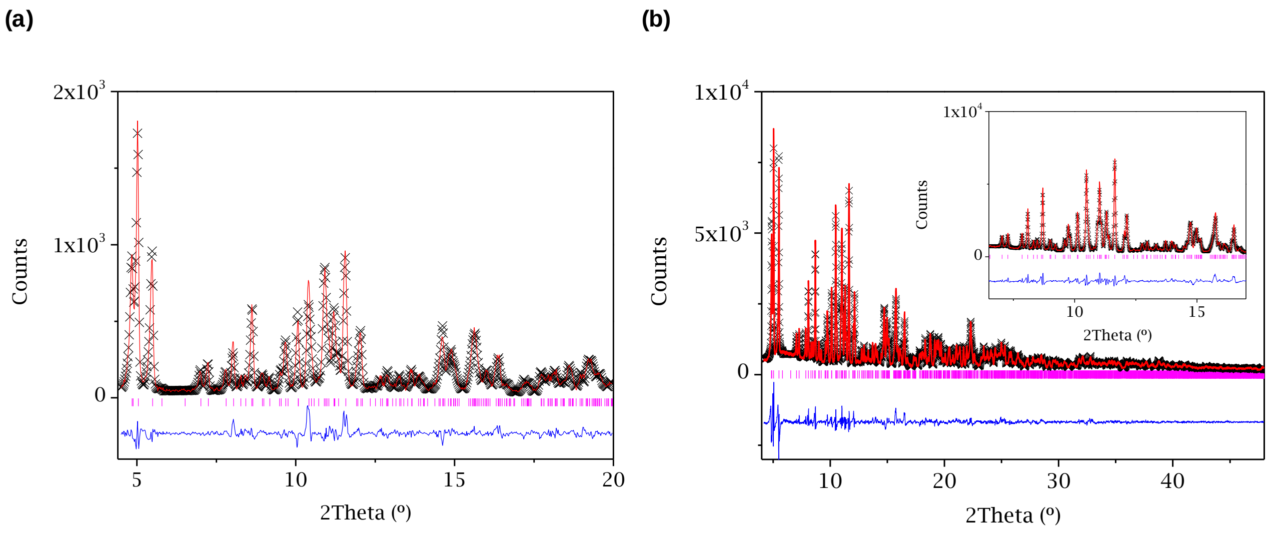


**Figure S15.** Rietveld plots for (a) **Eu-I-230** and (b) **Tb-I-230**.

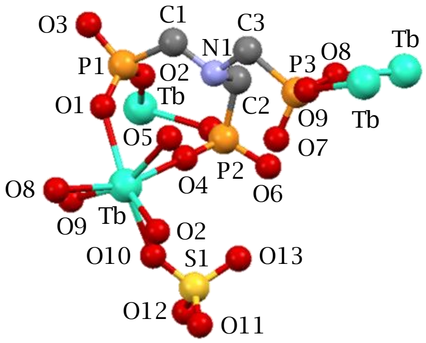


**Figure S16.** Coordination environment for **Eu-I-230**.

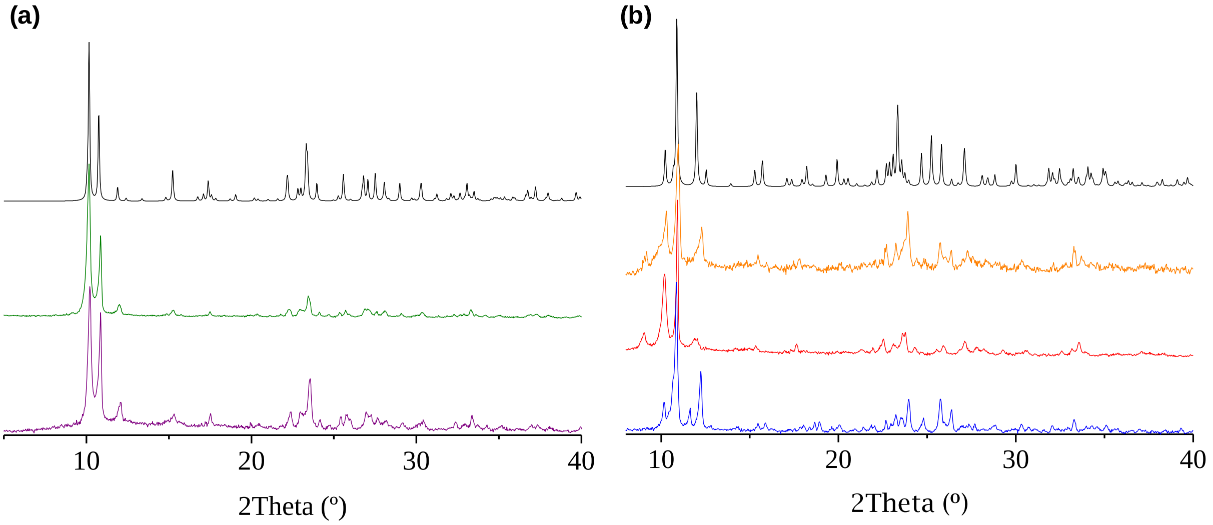


**Figure S17.** XRPD patterns for: (a) **SD-Pr-I** (olive) and **SD-Nd-I** (purple) compared with $[La_2(H_4NMP)_2(H_2O)_3(SO_4)]\cdot 6H_2O$ (black; CCDC no. 1496872); (b) **SD-Gd-I** (orange), **SD-Eu-I** (red) and **SD-Tb-I** (blue) compared with $[La_2(H_4NMP)_2(H_2O)_3(SO_4)]\cdot 2H_2O$ (black; CCDC no. 1496873).

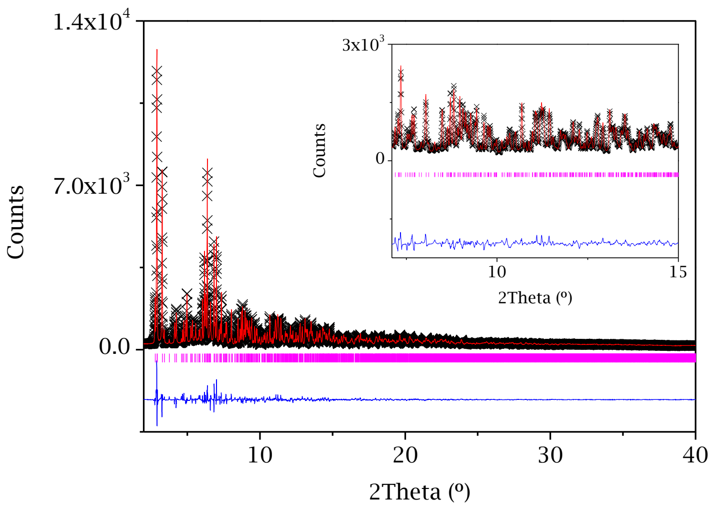


**Figure S18.** Rietveld plot for **SD-Tb-I**.

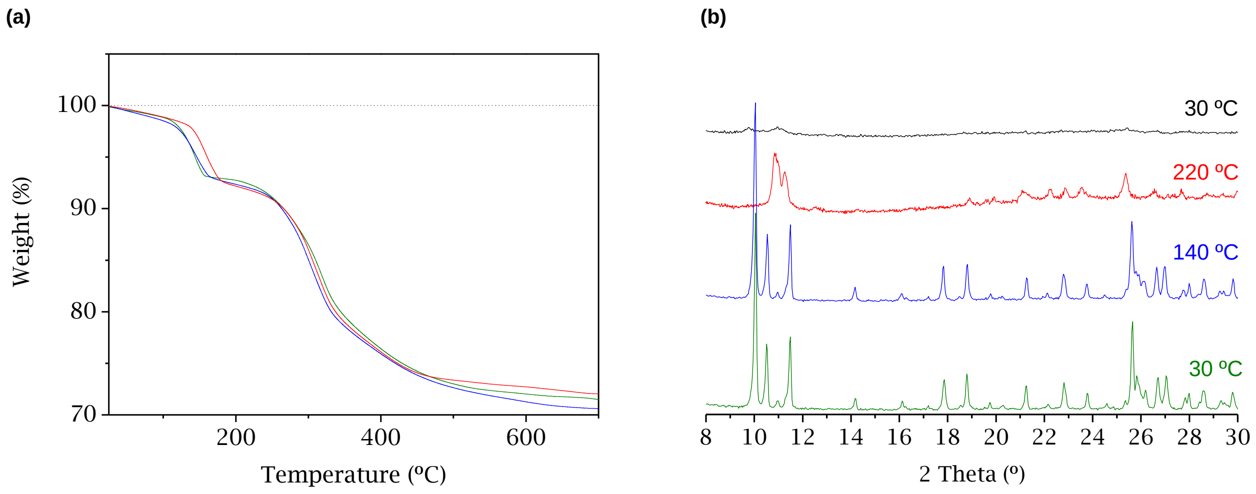


**Figure S19.** (a) TG curves for **Pr-II** (olive), **Eu-II** (red) and **Tb-II** (blue); (b) thermodiffractometric study for **Tb-II**.

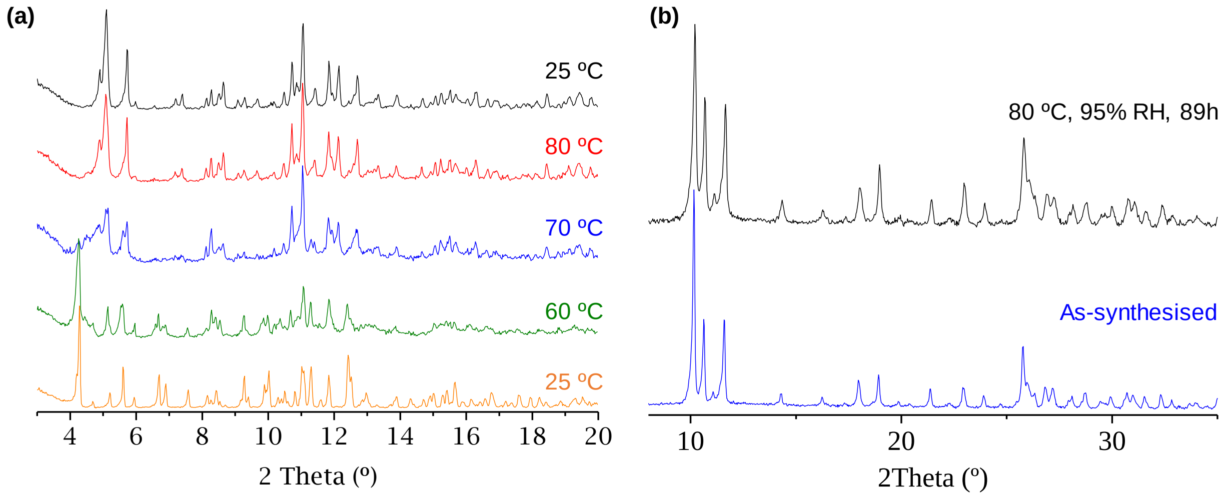


**Figure S20.** Thermodiffractometric studies at high relative humidity (95 %) for: (a) **Tb-I** and (b) **Tb-II**.

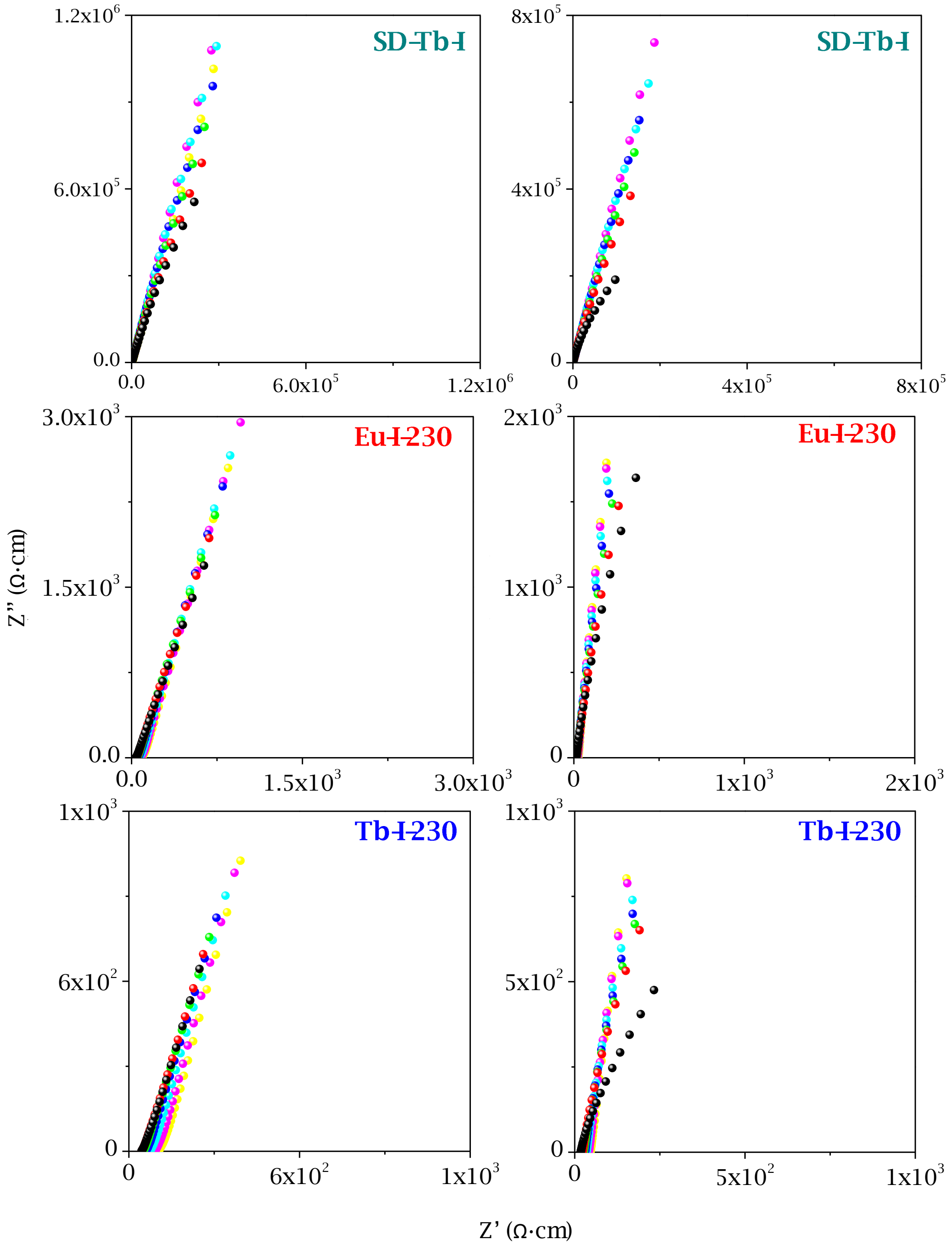


**Figure S21.** Complex impedance plane plots for **SD-Tb-I** and **Ln-I-230** derivatives at 75 (left) or 95% RH (right) and different temperatures: 80 (black), 70 (red), 60 (green), 50 (blue), 40 (cyan), 30 (magenta) and 25 °C (yellow).

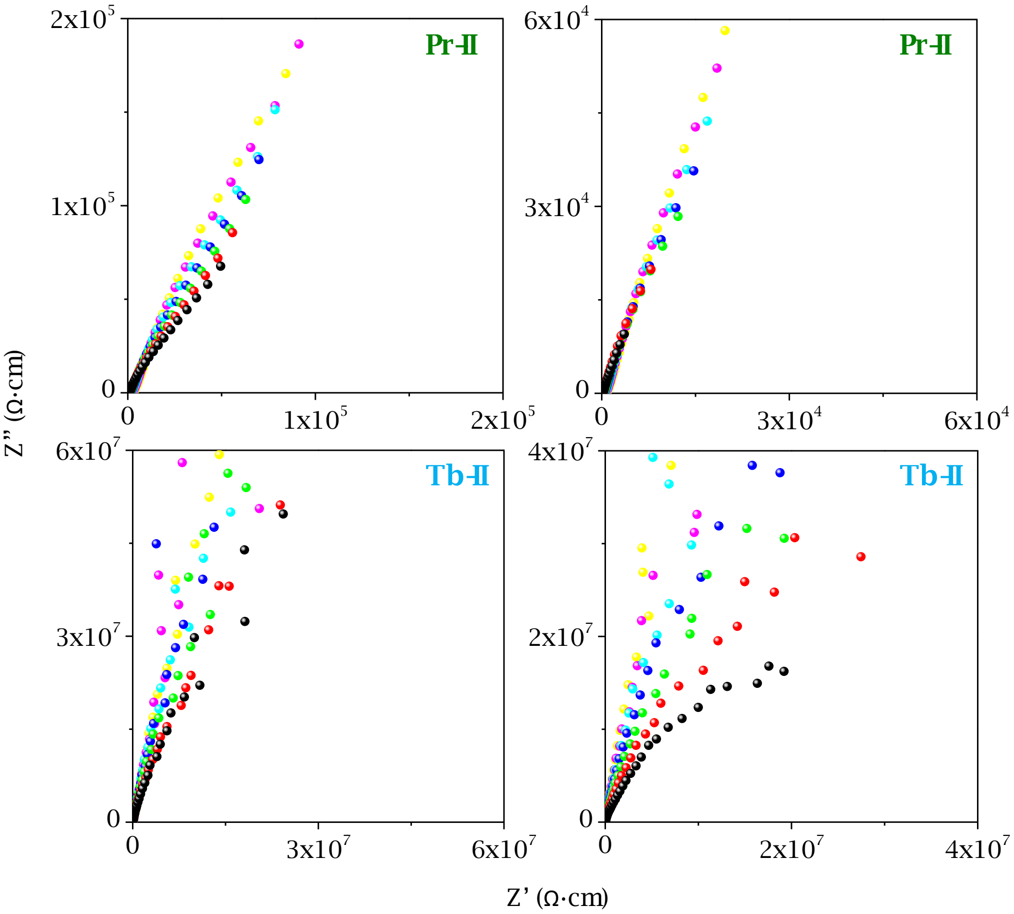


**Figure S22.** Complex impedance plane plots for **Series II** derivatives at 75 (left) or 95% RH (right) and different temperatures: 80 (black), 70 (red), 60 (green), 50 (blue), 40 (cyan), 30 (magenta) and 25 °C (yellow).

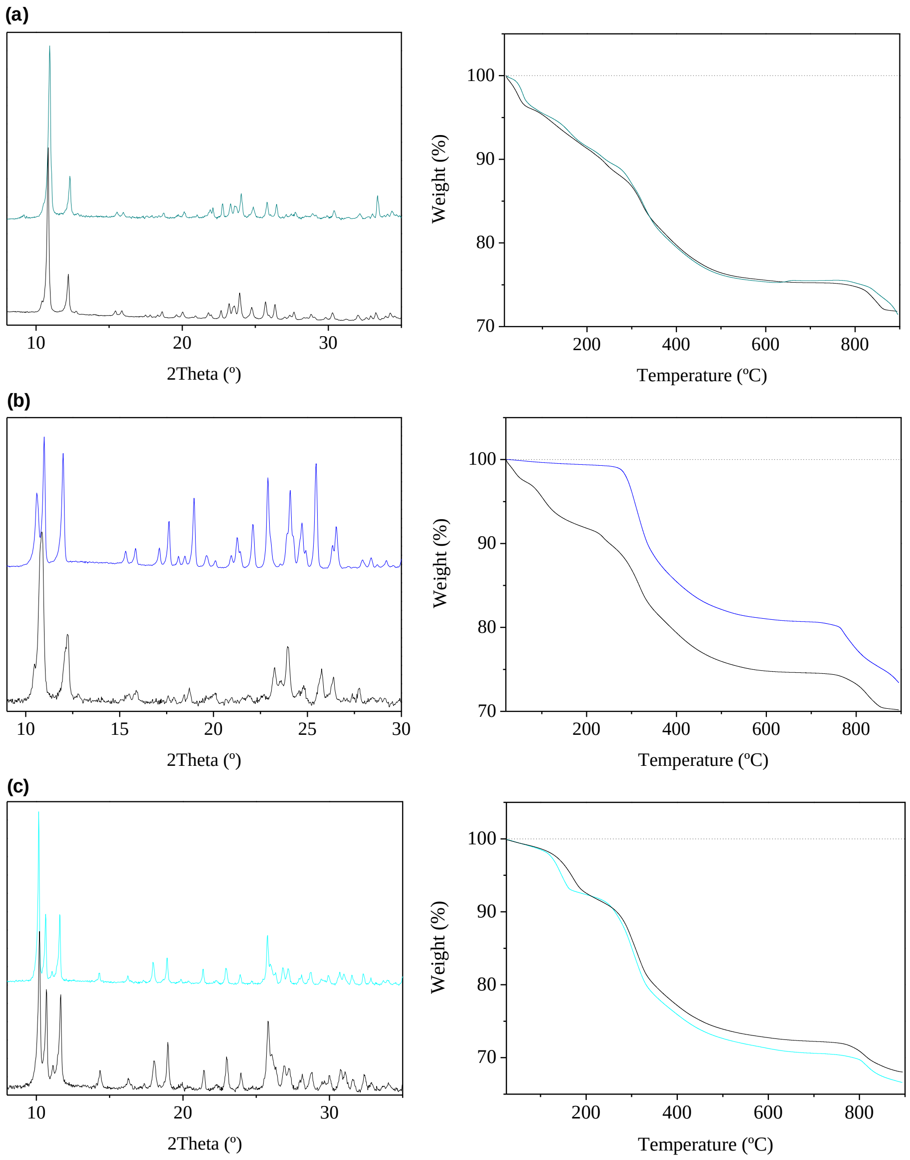


**Figure S23.** X-ray powder diffraction patterns (left) and thermal analysis (right) before (blue) and after (black) proton conductivity measurements for (a) **SD-Tb-I**, (b) **Tb-I-230** and (c) **Tb-II**.

(a)

(b)

(c)

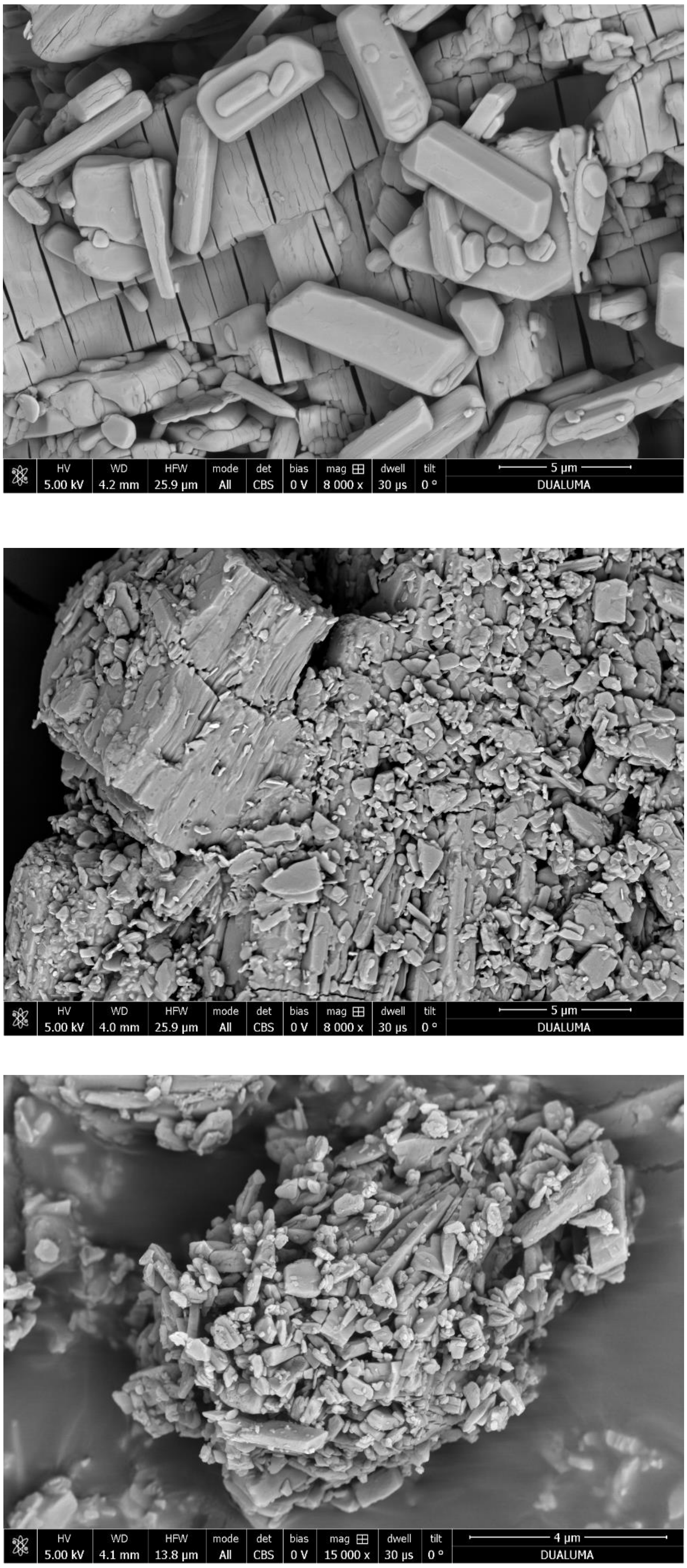


**Figure S24.** SEM images of (a) **Eu-I**, (b) **SD-Eu-I** and (c) **Tb-II**.

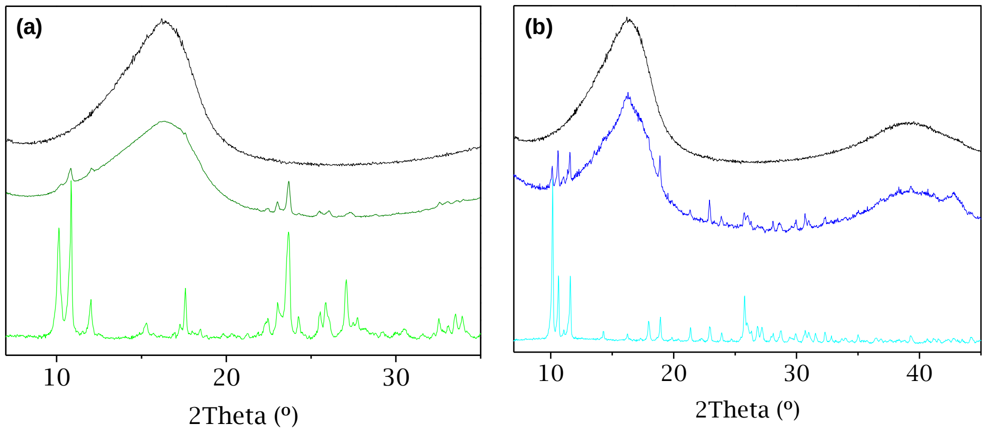


**Figure S25.** XRPD patterns of (a) **SD-Eu-I** (green), **N/SD-Eu-I** composite membrane (olive) and Nafion® membrane (black) and (b) **Tb-II** (cyan), **N/Tb-II** composite membrane (blue) and Nafion® membrane (black).

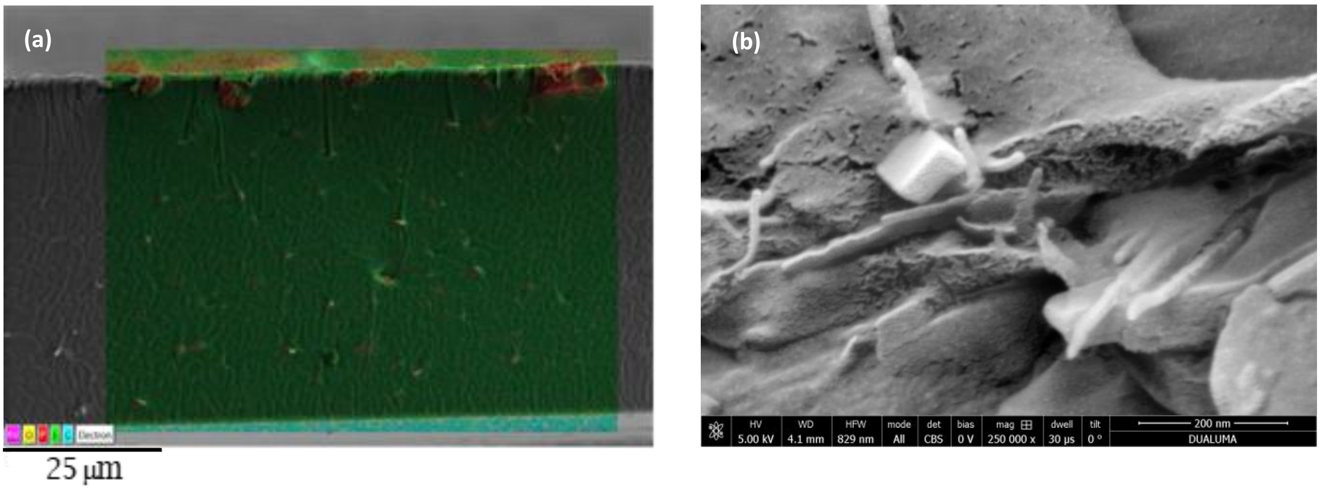


**Figure S26.** Cross-section SEM-EDX (a) and surface FE-SEM (b) images for **N/SD-Eu-I**.

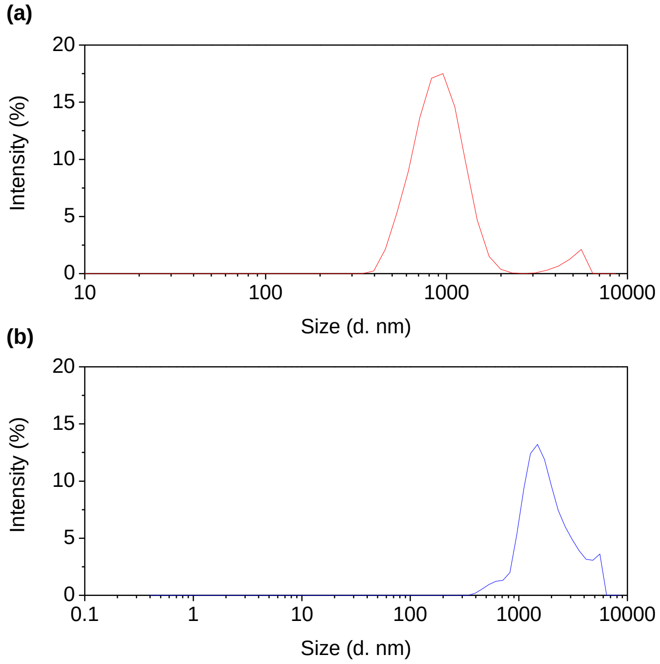


**Figure S27.** Particle size distribution for (a) **SD-Eu-I** and (b) **Tb-II**.

**Table S1**. Elements and partial DDEC6 charges range used for the MD simulations.

| Element | Charge range |
|---|---|
| Tb | +1.92 |
| S | +1.44 |
| P | +1.34, +1.39 |
| O | -0.62, -0.88 |
| N | +0.14 |
| C | -0.42, -0.43 |
| H | +0.16, +0.44 |

**Table S2**. Force field parameters (Lennard Jones parameters) used in the simulations.

| $\varepsilon$ | $\sigma_{LJ}$ | |
|---|---|---|
| 0.007000 | 3.074491 | # Tb8+3 UFF |
| 0.250000 | 3.550000 | # S_sulfate OPLS |
| 0.200000 | 3.740000 | # P_Phosphonate OPLS |
| 0.210000 | 2.960000 | # O_freewater OPLS |
| 0.000000 | 0.000000 | # H_freewater OPLS |
| 0.170000 | 2.960000 | # O_Sulfate OPLS |
| 0.000001 | 1.000000 | # H_Sulfate -> protons |
| 0.066000 | 3.500000 | # C_AminoPhosphonate OPLS |
| 0.170000 | 3.250000 | # N_3 OPLS |
| 0.030000 | 2.500000 | # H_Carbon OPLS |
| 0.030000 | 2.500000 | # H_Nitrogen OPLS |
| 0.000001 | 1.000000 | # H_Phosphonate ->Protons |
| 0.000000 | 0.000000 | # H_coordinatedwater OPLS |
| 0.210000 | 2.960000 | # O_P1 OPLS |
| 0.210000 | 2.960000 | # O_P2 OPLS |
| 0.210000 | 2.960000 | # O_P3 OPLS |
| 0.210000 | 2.960000 | # O_P4 OPLS |
| 0.210000 | 2.960000 | # O_P5 OPLS |
| 0.210000 | 2.960000 | # O_P6 OPLS |
| 0.210000 | 2.960000 | # O_coordWater1 OPLS |
| 0.210000 | 2.960000 | # O_coordWater2 OPLS |
| 0.210000 | 2.960000 | # O_Phosphonate_Non-coordinated OPLS |

*Bond Coeffs #(crystallographic length but DFT coefficients)*

| | | | |
|---|---|---|---|
| 1 | 225.543540 | 2.235678 | # Tb8+3 - O_Phosphonate UFF |
| 2 | 225.543540 | 2.235678 | # Tb8+3 - O_water UFF |
| 3 | 441.180925 | 1.521990 | # S-O DFT |
| 4 | 448.076090 | 1.690874 | # P-O DFT |
| 5 | 177.484490 | 1.451071 | # C-N DFT |
| 6 | 418.750210 | 1.044419 | # N-H DFT |
| 7 | 361.866870 | 1.109401 | # C-H DFT |
| 8 | 143.884200 | 1.812946 | # C-P DFT |
| 9 | 575.569900 | 0.963020 | # O-H free water DFT |
| 10 | 559.995206 | 0.990254 | # O-H coordinated water DFT |
| 11 | 520.916640 | 0.990254 | # O-H Phosphonate DFT ->Not Used |
| 12 | 572.809400 | 0.963570 | # O-H Sulfate -> DFT Not Used |

**Table S2**. Force field parameters (Lennard Jones parameters) used in the simulations (Continuation).

*Angle Coeffs #(crystallographic angles but DFT/UFF coefficients)*

| | | | |
|---|---|---|---|
| 1 | 166.732611 | 70.900000 | # Ow-Tb-Ow UFF |
| 2 | 122.882000 | 112.333333 | # O-P-O DFT |
| 3 | 118.267070 | 105.236666 | # O-P-C DFT |
| 4 | 122.882000 | 112.333333 | # O-P-O DFT |
| 5 | 118.267070 | 105.236666 | # O-P-C DFT |
| 6 | 276.209362 | 154.115000 | # Tb-O-P UFF |
| 7 | 166.732611 | 109.600000 | # Tb-O-H UFF |
| 8 | 1.00000000 | 107.060000 | # P-O-H -> Not used |
| 9 | 44.4964000 | 105.470000 | # H-O-H DFT |
| 10 | 65.2091600 | 108.580000 | # H-N-C DFT |
| 11 | 188.163000 | 110.333333 | # C-N-C DFT |
| 12 | 162.473930 | 118.000000 | # N-C-P DFT |
| 13 | 85.2589000 | 108.740000 | # N-C-H DFT |
| 14 | 62.7846200 | 107.770000 | # P-C-H DFT |
| 15 | 38.6943400 | 107.630000 | # H-C-H DFT |
| 16 | 140.000000 | 109.059760 | # O-S-O DFT |
| 17 | 140.000000 | 109.059760 | # O-S-O DFT |
| 18 | 137.883240 | 104.092280 | # S-O-H -> Not used |
| 19 | 166.732611 | 144.710000 | # O(P1)-Tb-O(P2) UFF |
| 20 | 166.732611 | 142.960000 | # O(P1)-Tb-O(P3) UFF |
| 21 | 166.732611 | 91.290000 | # O(P1)-Tb-O(P4) UFF |
| 22 | 166.732611 | 79.010000 | # O(P1)-Tb-O(P5) UFF |
| 23 | 166.732611 | 76.050000 | # O(P1)-Tb-O(P6) UFF |
| 24 | 166.732611 | 93.050000 | # O(P1)-Tb-O(POw1) UFF |
| 25 | 166.732611 | 72.910000 | # O(P1)-Tb-O(POw2) UFF |
| 26 | 166.732611 | 72.240000 | # O(P2)-Tb-O(P3) UFF |
| 27 | 166.732611 | 83.640000 | # O(P2)-Tb-O(P4) UFF |
| 28 | 166.732611 | 129.750000 | # O(P2)-Tb-O(P5) UFF |
| 29 | 166.732611 | 127.490000 | # O(P2)-Tb-O(P6) UFF |
| 30 | 166.732611 | 74.290000 | # O(P2)-Tb-O(POw1) UFF |
| 31 | 166.732611 | 71.830000 | # O(P2)-Tb-O(POw2) UFF |
| 32 | 166.732611 | 91.450000 | # O(P3)-Tb-O(P4) UFF |
| 33 | 166.732611 | 67.810000 | # O(P3)-Tb-O(P5) UFF |
| 34 | 166.732611 | 78.450000 | # O(P3)-Tb-O(P6) UFF |
| 35 | 166.732611 | 104.040000 | # O(P3)-Tb-O(POw1) UFF |
| 36 | 166.732611 | 143.690000 | # O(P3)-Tb-O(POw2) UFF |
| 37 | 166.732611 | 68.470000 | # O(P4)-Tb-O(P5) UFF |
| 38 | 166.732611 | 140.280000 | # O(P4)-Tb-O(P6) UFF |
| 39 | 166.732611 | 147.310000 | # O(P4)-Tb-O(POw1) UFF |
| 40 | 166.732611 | 79.5400000 | # O(P4)-Tb-O(POw2) UFF |
| 41 | 166.732611 | 72.170000 | # O(P5)-Tb-O(P6) UFF |
| 42 | 166.732611 | 144.060000 | # O(P5)-Tb-O(POw1) UFF |
| 43 | 166.732611 | 136.520000 | # O(P5)-Tb-O(POw2) UFF |
| 44 | 166.732611 | 71.900000 | # O(P6)-Tb-O(POw1) UFF |
| 45 | 166.732611 | 129.340000 | # O(P6)-Tb-O(POw2) UFF |

*Dihedral Coeffs*

| | | | |
|---|---|---|---|
| 1 | 0.136931 | 1 | 2 # O-P-O-Tb UFF |
| 2 | 0.070473 | 1 | 3 # O-P-C-N UFF |
| 3 | 0.091287 | 1 | 2 # C-P-O-Tb UFF |
| 4 | 0.030516 | 1 | 3 # H-N-C-P UFF |
| 5 | 0.230489 | 1 | 2 # O-S-O-H UFF ->Not Used |

**Table S3.** Calculated proton conductivity values from the MSD.

| T [°C] | σ [S · cm$^{-1}$] |
|---|---|
| 35 | 1.4 x $10^{-3}$ |
| 100 | 1.3 x $10^{-2}$ |
| 150 | 5.0 x $10^{-2}$ |

**Table S4.** Extrapolated low temperature data using the Arrhenius equation for the conductivity in the range 20-80 °C.

| T [°C] | σ [S · cm$^{-1}$] |
|---|---|
| 20 | 6.5 x $10^{-4}$ |
| 30 | 1.0 x $10^{-3}$ |
| 40 | 1.6 x $10^{-3}$ |
| 50 | 2.3 x $10^{-3}$ |
| 60 | 3.4 x $10^{-3}$ |
| 70 | 4.9 x $10^{-3}$ |
| 80 | 6.8 x $10^{-3}$ |

**Table S5.** Crystallographic data for compounds of **Series I** and **II.**

| **Phase** | **Pr-I#** | **Nd-I** | **Sm-I** | **Eu-I** | **Gd-I** | **Er-I** | **Yb-I** |
|---|---|---|---|---|---|---|---|
| Space Group | P $2_1$/c | P $2_1$/c | P $2_1$/c | P $2_1$/c | P $2_1$/c | P $2_1$/c | P $_2$1/c |
| Chemical formula | $C_6H_{38}N_2O_{34}P_6S_2Pr_2$ | $C_6H_{38}N_2O_{34}P_6S_2Nd_2$ | $C_6H_{38}N_2O_{34}P_6S_2Sm_2$ | $C_6H_{38}N_2O_{34}P_6S_2Eu_2$ | $C_6H_{38}N_2O_{34}P_6S_2Gd_2$ | $C_6H_{40}N_2O_{35}P_6S_2Er_2$ | $C_6H_{40}N_2O_{35}P_6S_2Yb_2$ |
| Formula mass (g·mol$^{-1}$) | 1228.13 | 608.39 | 596.33 | 1236.26 | 1246.83 | 1284.87 | 1296.43 |
| λ (Å) | 1.5418 | 0.71075 | 0.71073 | 0.7093 | 0.4124 | 0.7093 | 0.7093 |
| a (Å) | 8.5327(2) | 8.5197(2) | 8.6060(3) | 8.4993(1) | 8.5086(1) | 8.4562(2) | 8.4291(3) |
| b (Å) | 18.8189(5) | 19.1826(4) | 19.5335(7) | 19.4416(3) | 19.4894(4) | 21.3849(9) | 21.4041(12) |
| c (Å) | 10.7340(3) | 10.7060(3) | 10.6850(4) | 10.6096(1) | 10.6123(1) | 10.3316(2) | 10.2876(3) |
| β (°) | 109.172(2) | 109.264(3) | 109.853(4) | 109.5397(9) | 109.5674(7) | 109.7112(15) | 109.7420(19) |
| V (Å$^3$) | 1628.03(8) | 1651.71(8) | 1689.45(11) | 1652.18(5) | 1658.19(6) | 1758.85(12) | 1746.97(15) |
| Z | 2 | 2 | 2 | 2 | 2 | 2 | 2 |
| T (K) | 298 | 293(2) | 293(2) | 298 | 298 | 298 | 298 |
| Range data (°) | 4.00-69.98 | 2.12-27.46 | 3.27-29.33 | 3.70-50.00 | 2.00-20.00 | 4.03-42.01 | 4.30-42.00 |
| Independent Reflections | - | 3599 | 3637 | 2930 | 1032 | 1907 | 1888 |
| Data / restrains / parameters | - | 3781/1/170 | 4360/0/222 | 4676/48/133 | 3000/49/111 | 3834/65/126 | 3834/66/129 |
| $R_{WP}$ | - | - | - | 0.0821 | 0.0720 | 0.1326 | 0.1209 |
| $R_P$ | - | - | - | 0.0631 | 0.0561 | 0.1000 | 0.0905 |
| $R_F$ | - | - | - | 0.0300 | 0.0198 | 0.0587 | 0.0450 |
| R factor [I > 2σ(I)] | - | $^aR_1$= 0.0439; $^awR_2$= 0.1193 | $^aR_1$= 0.0884; $^awR_2$= 0.2014 | - | - | - | - |
| R factor (all data) | - | $^aR_1$= 0.0461; $^awR_2$= 0.1213 | $^aR_1$= 0.1070; $^awR_2$= 0.2104 | - | - | - | - |
| GoF | - | 1.108 | 1.138 | - | - | - | - |
| **CCDC number* | - | 2003913 | 2003912 | 1980603 | 1980602 | 1980605 | 1980606 |

# Le Bail fit.

$^aR_1(F) = \Sigma||F_o| - |F_c||/\Sigma|F_o|$; $wR_2(F^2) = [\Sigma w(F_o^2 - F_c^2)^2/\Sigma F^4]^{1/2}$.

*CCDC contains the supplementary crystallographic data for this paper. These data can be obtained free of charge via www.ccdc.cam.ac.uk/data_request/cif, or by emailing data_request@ccdc.cam.ac.uk, or by contacting The Cambridge Crystallographic Data Centre, 12 Union Road, Cambridge CB2 1EZ, UK; fax: +44 1223 336033.

**Table S5.** Crystallographic data for compounds of **Series I** and **II** (Continuation).

| **Phase** | **Pr-II** | **Nd-II#** | **Eu-II#** | **Gd-II** | ***Eu-I-230*** |
|---|---|---|---|---|---|
| Space Group | P -1 | P -1 | P -1 | P -1 | *P 2₁/c* |
| Chemical formula | $C_3H_{15}NO_{15}P_3SPr$ | $C_3H_{15}NO_{15}P_3SNd$ | $C_3H_{15}NO_{15}P_3SEu$ | $C_3H_{15}NO_{15}P_3SGd$ | $C_6H_{22}N_2O_{26}P_6Eu_2S_2$ |
| Formula mass (g·mol$^{-1}$) | 571.04 | 574.38 | 582.10 | 587.39 | 1092.14 |
| λ (Å) | 1.5406 | 1.5418 | 1.5418 | 0.7093 | 0.7093 |
| a (Å) | 10.1779(4) | 10.1853(5) | 10.1156(3) | 10.1038(5) | 8.887(1) |
| b (Å) | 9.6687(5) | 9.6698(5) | 9.6128(2) | 9.6041(4) | 16.166(3) |
| c (Å) | 9.5553(4) | 9.5686(5) | 9.4575(2) | 9.4144(4) | 10.480(1) |
| α (°) | 110.7290(35) | 110.565(4) | 110.760(2) | 110.6369(26) | - |
| β (°) | 111.7803(30) | 111.900(3) | 111.555(2) | 111.6729(24) | 109.95(1) |
| γ (°) | 101.453(4) | 101.472(4) | 101.470(2) | 101.5449(33) | - |
| V (Å$^3$) | 755.45(5) | 757.35(6) | 740.34(3) | 734.96(5) | 1415.1(6) |
| Z | 2 | 2 | 2 | 2 | 2 |
| T (K) | 298 | 298 | 298 | 298 | 298 |
| Range data (°) | 9.56-89.98 | 4.00-69.98 | 8.00-69.98 | 3.50-65.00 | 2.99-19.97 |
| M(20) | - | 14 | 14.4 | - | - |
| F(20) | - | 29 | 26.4 | - | - |
| Independent Reflections | 1223 | - | - | 5353 | 176 |
| Data / restrains / parameters | 4787/57/119 | - | - | 6195/46/119 | 774/49/111 |
| $R_{WP}$ | 0.1184 | - | - | 0.1213 | 0.1046 |
| $R_P$ | 0.0830 | - | - | 0.0897 | 0.0798 |
| $R_F$ | 0.0683 | - | - | 0.0291 | 0.0467 |
| *CCDC number | 2003908 | - | - | 2003909 | 2003910 |

# Le Bail fit.

*CCDC contains the supplementary crystallographic data for this paper. These data can be obtained free of charge via www.ccdc.cam.ac.uk/data_request/cif, or by emailing data_request@ccdc.cam.ac.uk, or by contacting The Cambridge Crystallographic Data Centre, 12 Union Road, Cambridge CB2 1EZ, UK; fax: +44 1223 336033.

**Table S6.** H-bond distances for **Tb-I**.

| **D-H···A** | **D-H (Å)** | **H···A (Å)** | **D···A (Å)** | **D-H···A (°)** |
|---|---|---|---|---|
| O8-H8···Ow4 | 0.98 | 1.66 | 2.641(4) | 173.3 |
| Ow4-Hw4A···O12[#1] | 0.85 | 1.99 | 2.823(5) | 167.1 |
| Ow4-Hw4B···O3[#2] | 0.85 | 2.16 | 2.951(4) | 154.4 |
| Ow2-Hw2A···O11[#3] | 0.86 | 1.96 | 2.766(4) | 154.7 |
| Ow2-Hw2B···O1[3#4] | 0.86 | 1.87 | 2.672(4) | 153.8 |
| O2-H2···O11[#3] | 0.82 | 1.80 | 2.560(4) | 154.0 |
| O5-H5···Ow3 | 0.82 | 1.88 | 2.691(5) | 170.1 |
| Ow3-Hw3A··· O9[#4] | 0.85 | 2.11 | 2.901(4) | 153.7 |
| Ow3-Hw3B···Ow2[#5] | 0.85 | 2.24 | 3.005(5) | 149.3 |
| Ow1-Hw1A···O12[#6] | 0.86 | 2.20 | 2.982(4) | 151.2 |
| Ow1-Hw1B···Ow3[#7] | 0.86 | 2.14 | 2.812(4) | 133.9 |
| N1-H1···O4[#8] | 0.98 | 2.06 | 2.938(4) | 148.7 |
| O10-H10···O9 | 0.82 | 1.71 | 2.511(4) | 166.2 |
| C1-H1A···O13[#9] | 0.97 | 2.29 | 3.104(5) | 140.5 |

#1 -x+2, -y+1, -z+1. #2 x+1, y, z. #3 x-1, -y+1/2, z-1/2. #4 -x+1, y-1/2, -z+3/2. #5 -x, -y, -z+1. #6 x-2, -y+1/2, z-1/2. #7 x-1, y, z. #8 x, -y+1/2, z-1/2. #9 -x+1, -y+1, -z+1.

**Table S7.** H-bond distances for **Pr-I***.

| D-H···A | D-H (Å) | H···A (Å) | D···A (Å) | D-H···A (°) |
|---|---|---|---|---|
| O7-H7···O16 | 0.84 | 1.78 | 2.594(8) | 161.7 |
| O7-H7···O14B[#1] | 0.84 | 1.78 | 2.498(15) | 142.6 |
| O4-H4···O20A[#2] | 0.84 | 1.84 | 2.643(6) | 158.7 |
| O1-H1···S2[#1] | 0.84 | 2.59 | 3.114(6) | 122.0 |
| O1-H1···O15 | 0.84 | 1.93 | 2.744(8) | 161.9 |
| O1-H1···O14B[#1] | 0.84 | 1.98 | 2.681(17) | 139.9 |
| N1-H1A···O3 | 1.00 | 2.07 | 2.938(5) | 143.6 |
| O3-H3B··· O17[#4] | 0.99 | 2.41 | 3.092(9) | 125.1 |
| C1-H1C···O8 | 0.99 | 2.34 | 3.184(5) | 143.1 |
| O11A-H11A···O16[#5] | 0.98 | 1.91 | 2.855(14) | 162.6 |
| O11A-H11B···O17[#6] | 0.99 | 2.08 | 2.738(13) | 122.7 |
| O19-H19A···O5[#7] | 0.87 | 2.04 | 2.794(5) | 144.3 |
| O19-H19B···O9[#8] | 0.87 | 2.19 | 2.930(5) | 142.7 |
| O10A-H10A···O15 | 0.98 | 1.75 | 2.726(11) | 171.6 |
| O20A-H20A···O19 | 0.87 | 1.84 | 2.669(6) | 157.5 |
| O20B-H20D···O19 | 0.87 | 2.06 | 2.892(18) | 158.6 |

#1 x+1, y, z; #2 -x+1, y-1/2, -z+1/2; #3 x, -y+1/2, z+1/2; #4 -x+2, y-1/2, -z+3/2; #5 x-1, y, z; #6 -x+1, -y+1, -z+1; #7 x, -y+1/2, z-1/2; #8 x-1, -y+1/2, z-1/2.

**Table S8.** H-bond distances for **Tb-II**.

| D-H···A | D···A (Å) | D-H···A | D···A (Å) |
|---|---|---|---|
| O1···O8 | 3.115(31) | O8···O12 | 3.475(27) |
| O1···O11 | 2.896(30) | O9···O19 | 2.18(4) |
| O2···Ow2 | 2.77(4) | O9···Ow2 | 3.18(4) |
| O3···O12 | 3.23(4) | O10···O10 | 2.79(5) |
| O3···Ow2 | 2.491(28) | O11···Ow1 | 3.00(4) |
| O4···Ow1 | 2.93(4) | Ow1···Ow1 | 2.97(6) |
| O4···Ow2 | 3.03(4) | Ow2···Ow2 | 2.96(6) |
| O7···Ow1 | 2.67(4) | | |

**Table S9.** H-bond distances for **Tb-I-230**.

| D-H···A | D···A (Å) | D-H···A | D···A (Å) |
|---|---|---|---|
| O1···O12 | 2.565(29) | O6···O7 | 2.904(25) |
| O3···O6 | 3.195(23) | O6···O11 | 3.083(20) |
| O3···O10 | 2.758(27) | O6···O13 | 2.873(26) |
| O3···O11 | 2.894(28) | O7···O11 | 2.413(27) |
| O3···O12 | 3.053(26) | O9···O10 | 2.957(24) |

**Table S10.** H-bond distances for **SD-Tb**.

| D-H···A | D···A (Å) | D-H···A | D···A (Å) |
|---|---|---|---|
| O1W···O3 | 2.902(20) | O7···O3W | 2.679(11) |
| O1W···O6 | 3.178(22) | O10···O3 | 3.131(31) |
| O1W···O7 | 3.193(22) | O11···Ow | 2.536(29) |
| O1W···O11 | 2.535(20) | O11···O3W | 2.635(20) |
| O1W···O13 | 3.136(25) | O12···O3W | 2.501(22) |
| Ow···O2W | 3.12(4) | O12···O2W | 2.835(33) |
| O1···O2W | 3.016(23) | O13···Ow | 2.583(33) |
| O2···O3W | 2.793(21) | O13···Ow | 2.89(4) |
| O3···O11 | 2.858(26) | O13···O3W | 2.897(32) |
| O6···O12 | 2.551(24) | O13···O3W | 3.169(29) |
| O6···O13 | 2.411(24) | O3W···O5 | 3.055(23) |
| O7···O11 | 2.414(20) | O2W···O2W | 2.910(32) |

**References**

[1] M. Koichi, I. Fujio, VESTA: a three-dimensional visualization system for electronic and structural analysis. *J. Appl. Cryst.* **2008**, *41*, 653-658.

[2] Jmol: an open-source Java viewer for chemical structures in 3D. http://www.jmol.org/
